# Mitochondrial control of blood-brain barrier homeostasis in neuroinflammatory psychiatric disorders

**DOI:** 10.3389/fnmol.2026.1924581

**Published:** 2026-08-24

**Authors:** Larli Misael Pérez-López, Alberto Camacho-Morales, Ivón Howland-Álvarez, Antonio Alí Pérez-Maya

**Affiliations:** 1 Department of Biochemistry and Molecular Medicine, Faculty of Medicine, Autonomous University of Nuevo León, Monterrey, Mexico; 2 Department of Biological Sciences, Faculty of Health Sciences, Technical University of Manabí, Portoviejo, Ecuador

**Keywords:** blood-brain barrier, immunometabolism, mitochondrial dysfunction, neuroinflammation, neurovascular unit, precision psychiatry, psychiatric disorders

## Abstract

Neuroinflammatory mechanisms are increasingly recognized in biologically defined subgroups of psychiatric disorders, yet the cellular interfaces linking peripheral immune activation to brain dysfunction remain incompletely understood. This review examines the blood-brain barrier (BBB) and neurovascular unit (NVU) as dynamic immunometabolic structures whose stability depends on mitochondrial bioenergetics, redox signaling, calcium handling, mitophagy, and innate immune regulation. We integrate human postmortem, neuroimaging, cerebrospinal fluid and circulating biomarker findings with mechanistic evidence from cellular and animal models to examine whether mitochondrial dysfunction may be associated with BBB vulnerability, endothelial activation, altered tight-junction organization, and neuroinflammatory signaling. We discuss how this mitochondrial-BBB axis may contribute to transdiagnostic phenotypes such as cognitive impairment, anhedonia, fatigue, negative symptoms, affective dysregulation, and treatment resistance. Candidate biomarkers, including inflammatory mediators, BBB permeability markers, mitochondrial DNA, bioenergetic readouts, mitophagy markers, and neuroimaging measures, are considered as tools for patient stratification rather than diagnosis. Finally, we evaluate therapeutic implications, including mitochondrial-targeted interventions, BBB-protective strategies, anti-inflammatory approaches, metabolic modulation, and precision psychiatry frameworks. We argue that the mitochondrial regulation of BBB homeostasis represents a promising but still emerging framework for understanding neuroinflammatory psychiatric phenotypes and designing biomarker-guided studies.

## 1 Introduction

Neuroinflammation has emerged as one of the most consistently reported biological dimensions across several psychiatric disorders. Evidence of both peripheral and central inflammatory profiles has been reported in schizophrenia (SZ), bipolar disorder (BD), major depressive disorder (MDD), post-traumatic stress disorder (PTSD), autism spectrum disorders (ASD), and generalized anxiety disorder (GAD) (Yuan et al., 2019; Miller, 2020). However, although inflammatory responses have been widely documented in psychiatric disorders, it is not uniformly reported in all patients. For instance, only 25%–30% of individuals diagnosed with major depression showed clearly elevated levels of pro-inflammatory cytokines, highlighting the marked biological heterogeneity that exists among patients (Osimo et al., 2019). Based on this variability, neuroinflammation is unlikely to represent a single underlying trait conferring susceptibility to psychiatric disorders. Instead, it appears to function as a biological dimension capable of influencing symptom severity, disease progression, and treatment response (Khandaker et al., 2017; Miller, 2020). In addition, growing evidence suggests that inflammation may be more closely associated with specific symptom domains, including anhedonia and cognitive impairment, regardless of the diagnostic category (Miller, 2020; Solomon et al., 2025).

The blood-brain barrier (BBB) is a highly dynamic neuroimmune interface that forms part of the neurovascular unit (NVU). This structure is composed of endothelial cells, pericytes, astrocytes, the basement membrane, perivascular microglia, and extracellular matrix (ECM) components (Corrigan et al., 2023; Konstantinou and Warsh, 2026). Beyond regulating the selective transport of molecules between the circulation and the central nervous system (CNS), the NVU actively contributes to immune surveillance, vascular signaling, and the maintenance of redox homeostasis (Konstantinou and Warsh, 2026). Experimental and neurological-disease studies indicate that loss of barrier integrity can alter communication between the peripheral immune system and the brain. Whether comparable changes contribute directly to psychiatric symptoms remains uncertain (Greene et al., 2020; Konstantinou and Warsh, 2026).

Once activated, peripheral inflammation might also increase the permeability of the BBB, altering cytokine transport systems, and engaging Toll-like receptors (TLRs) by damage-associated molecular patterns (DAMPs) and pathogen-associated molecular patterns (PAMPs) (Serna-Rodríguez et al., 2022). Accordingly, DAMPs-related neuroinflammation was detected in the hippocampus and prefrontal cortex (PFC) (Tynan et al., 2010; Liu L. L. et al., 2019; Xu et al., 2020), two brain regions implicated in the pathophysiology and progression of psychiatric disorders (Liu et al., 2017; Liu L. L. et al., 2019; Pandey et al., 2019). The current evidence suggests a bidirectional crosstalk between BBB permeability, NVU stability, and systemic pro-inflammatory recruitment into the brain contributing to the development and progression of psychiatric disorders.

In recent years, mitochondria have come to be recognized as more than organelles solely responsible for ATP production. They are now understood to participate in several processes essential for maintaining neurovascular homeostasis. These functions include the regulation of reactive oxygen species (ROS), calcium-dependent signaling, and pathways associated with apoptosis and cellular stress responses (Kim et al., 2019; Jones et al., 2021). Mitochondria also play an active role in innate immunity and inflammatory signaling, including activation of the NOD-, leucine-rich repeat-, and pyrin domain-containing protein 3 (NLRP3) inflammasome under conditions of cellular stress or damage (Jones et al., 2021; Nunes et al., 2025). In this context, mitochondrial function may be a key factor determining whether the NVU preserves physiological stability or shifts toward a pro-inflammatory and potentially neurotoxic state (Büttiker et al., 2023).

### 1.1 Search strategy and scope

This is a narrative and conceptual review. PubMed was searched for English-language articles published up to 25 July 2026. Three complementary strategies were used: (i) a core search addressing the mitochondrial–BBB/NVU axis; (ii) a psychiatric-disorder search; and (iii) a therapeutic search focused on mitochondria-targeted interventions. The complete Boolean strategies are reported in Supplementary material to support reproducibility. One author (LP-L) screened titles and abstracts and assessed potentially relevant full texts. Eligible sources included peer-reviewed mechanistic studies, human biomarker and neuroimaging studies, postmortem analyses, meta-analyses, translational studies, and experimental BBB models. Preclinical and non-psychiatric BBB-injury models were retained when they provided mechanistic evidence directly relevant to mitochondrial regulation of barrier integrity. Studies without relevant mechanistic or clinical content, abstracts with insufficient information, and reports unrelated to the review domains were excluded. Reference lists of eligible articles were also screened to identify additional sources. Because this was a narrative review, no formal risk-of-bias assessment, meta-analysis, or independent duplicate screening was performed.

## 2 The BBB and the NVU

### 2.1 Cellular architecture of the NVU

The NVU comprises neurons, astrocytes, microglia, endothelial cells, and pericytes and coordinates cerebral blood flow and BBB integrity (Muoio et al., 2014). Brain endothelial cells (BECs) form the physical barrier and have relatively high mitochondrial content, supporting barrier regulation beyond ATP production (Aragón-González et al., 2022; Wang Y. et al., 2023). Pericytes stabilize capillaries and share basement-membrane contacts with endothelial cells (Daneman et al., 2010), whereas astrocytic endfeet coordinate vascular metabolic support with neuronal activity. Perivascular microglia and vessel-associated macrophages monitor the neurovascular interface and, during inflammation, can release secreted phosphoprotein 1 and chemokines that promote phagocytic states and remodeling of perivascular synapses and astrocytic endfeet (De Schepper et al., 2023; Zhu et al., 2023). The basement membrane and ECM provide structural and signaling support (Zhang et al., 2023; Konstantinou and Warsh, 2026). Neurovascular coupling requires coordination among these components; although altered perfusion and coupling are associated with clinical abnormalities in psychiatric populations, a primary mitochondrial contribution remains unproven (Büttiker et al., 2023; Song et al., 2024).

### 2.2 Molecular determinants of BBB homeostasis

The homeostasis of the BBB depends on a highly integrated molecular network that regulates paracellular permeability, endothelial adhesion, selective transport, immune cell interactions, and ECM remodeling.

Paracellular permeability and endothelial adhesion: Among the most important structural components are tight junction proteins, particularly claudin-5, occludin, and zonula occludens-1 (ZO-1), which restrict the paracellular passage of solutes and help maintain endothelial polarity (Jiao et al., 2011). In addition, endothelial stability is reinforced by adherens junctions, particularly through vascular endothelial cadherin (VE-cadherin)-mediated interactions. While tight junctions mainly regulate paracellular diffusion, adherens junctions help preserve endothelial architecture and contribute to BBB maturation. Under inflammatory or angiogenic conditions, disruption of VE-cadherin signaling can promote endothelial retraction, loss of intercellular contact, and increased vascular permeability (Park et al., 2021).

Selective transport: The BBB also functions as a highly specialized transport interface. glucose transporter 1 (GLUT1) is responsible for glucose delivery to the brain and is essential for maintaining the energetic demands of the CNS (Veys et al., 2020); L-type amino-acid transporter 1 (LAT1) regulates the transport of essential amino acids and contributes to glutamine homeostasis within the brain interstitial space (Dolgodilina et al., 2016); In parallel, P-glycoprotein (P-gp) acts as a major efflux transporter that limits the accumulation of xenobiotics, metabolites, and pharmacological compounds within the brain (Chaves et al., 2024). Consequently, transporter dysfunction could reflect loss of barrier metabolic and pharmacological selectivity.

Immune cell interactions: During neuroinflammatory conditions, BECs can adopt an activated phenotype characterized by increased expression of adhesion molecules such as intercellular adhesion molecule 1 (ICAM-1) and vascular cell adhesion molecule 1 (VCAM-1). These molecules facilitate leukocyte adhesion, arrest, and transmigration into the CNS. Recent studies have shown that elevated ICAM-1 levels enhance interactions between T cells or peripheral blood mononuclear cells (PBMCs) and BBB models under flow conditions, whereas VCAM-1 appears to contribute directly to endothelial dysfunction in models of chronic cerebral hypoperfusion (Soldati et al., 2023).

Trophic signals derived from the NVU: The Wnt/β-catenin pathway is essential for the induction and maintenance of the brain endothelial phenotype, and loss of this signaling axis in adult endothelial cells compromises BBB integrity while promoting neuroinflammation (Liebner et al., 2008; Tran et al., 2016). Similarly, Sonic Hedgehog released by astrocytes supports BBB stability and promotes an immunologically quiescent environment (Alvarez et al., 2011). Transforming growth factor beta (TGF-β) signaling, particularly within the pericyte–endothelial axis, also contributes to vascular stabilization and barrier maintenance (Schumacher et al., 2023).

**FIGURE 1:**
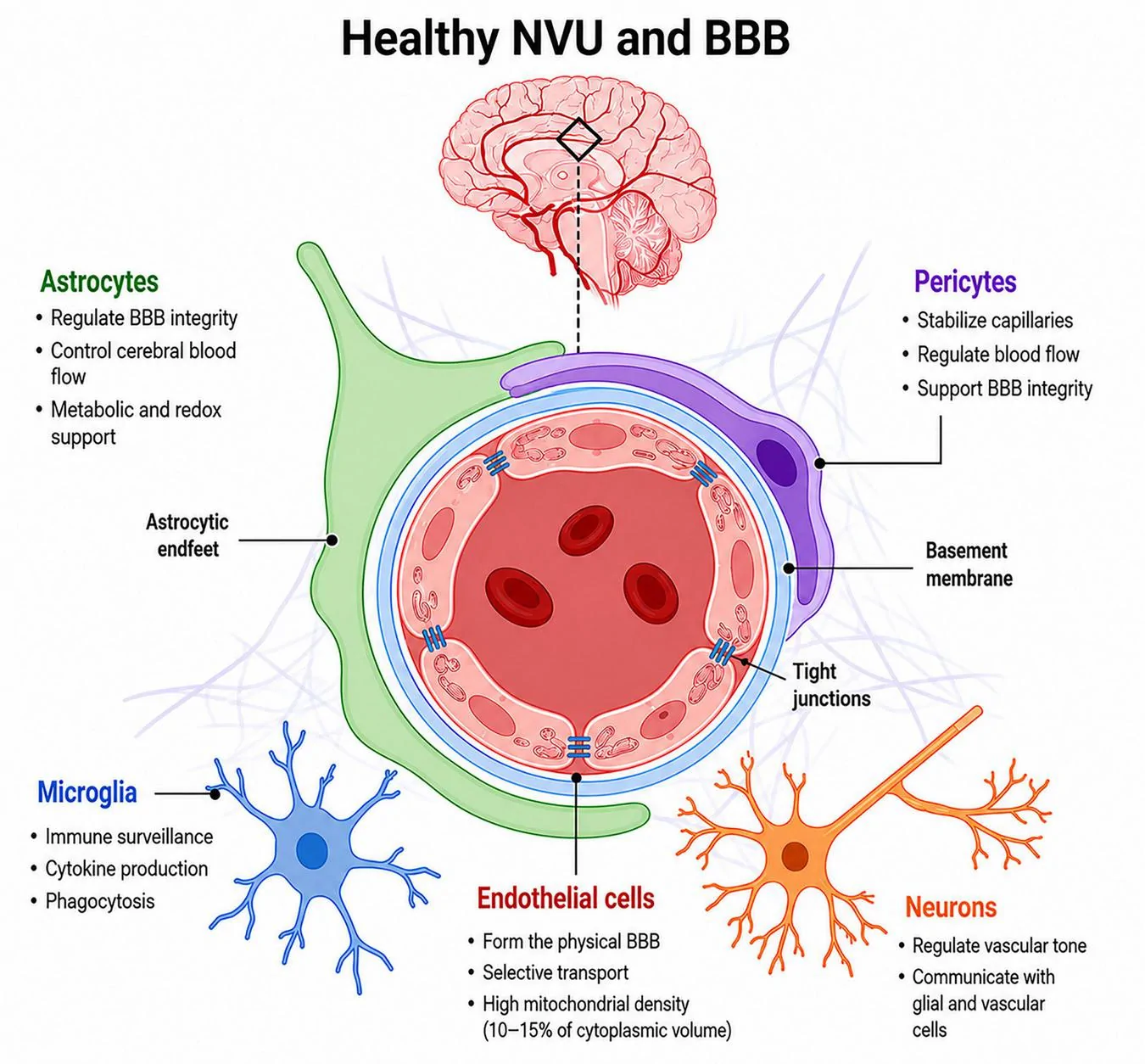
Cellular organization of the neurovascular unit (NVU) and functions relevant to blood-brain barrier (BBB) homeostasis. The cross-sectional cerebral capillary is formed by brain endothelial cells (BECs; red/pink), joined by tight junctions (blue), and surrounded by the basement membrane (light blue), astrocytic endfeet (green), pericytes (purple), microglia (blue), and neurons (orange). BECs provide selective transport and the physical barrier; astrocytes provide metabolic and redox support and help regulate cerebral blood flow; pericytes stabilize capillaries and support BBB maturation; microglia mediate immune surveillance; and neurons communicate with glial and vascular cells during neurovascular coupling. Black leader lines identify structures; the figure contains no activation or inhibition symbols. Color denotes cell identity and is retained consistently in Figures 2, 3. Cross-sectional cerebral capillary surrounded by a continuous basement membrane, green astrocytic endfeet, purple pericytes, blue microglia, and orange neurons. Red/pink endothelial cells form the vessel wall and are linked by blue tight junctions. Labels summarize the contribution of each neurovascular unit cell type to selective transport, capillary stability, cerebral blood-flow regulation, immune surveillance, and metabolic support under homeostatic conditions.

ECM remodeling: Vascular endothelial growth factor (VEGF), matrix metalloproteinases (MMPs), and ECM remodeling constitute another important regulatory axis within the BBB. Although VEGF is essential for angiogenesis and vascular repair, excessive VEGF signaling can increase BBB permeability (Valable et al., 2005). Likewise, MMP-2 and MMP-9 can degrade ECM components and tight junction proteins, thereby facilitating barrier opening under inflammatory, ischemic, or immune infiltrative conditions (Feng et al., 2011). Overall, current evidence indicates that BBB integrity relies on a delicate balance between tight junctions, transport systems, adhesion molecules, signaling pathways, and the ECM. The cellular components of the NVU and the functions relevant to BBB integrity are summarized in Figure 1.

## 3 Mitochondrial biology in the NVU and BBB control

### 3.1 Mitochondrial functions beyond ATP production

**FIGURE 2:**
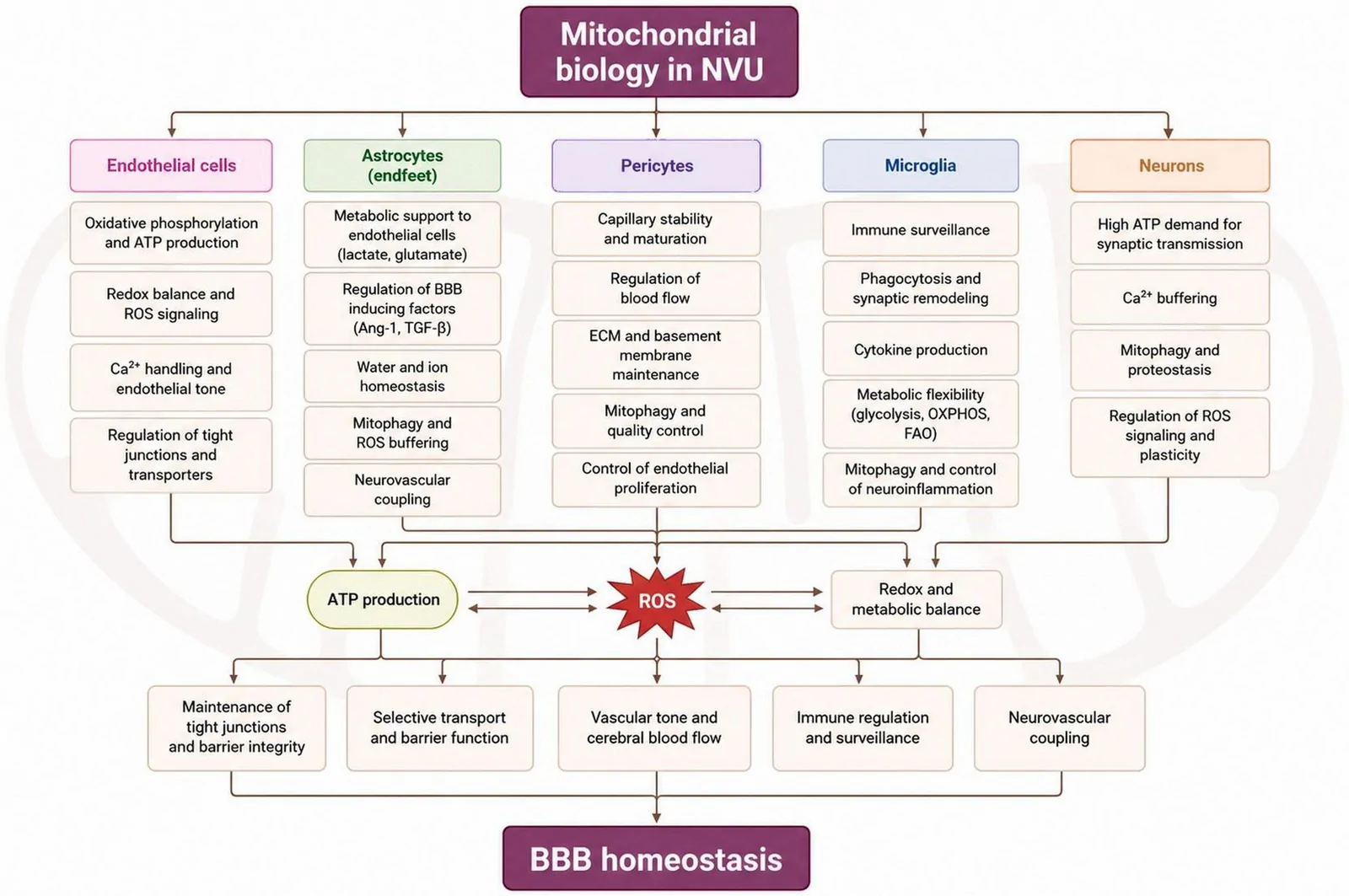
Cell-specific mitochondrial functions that support NVU and BBB homeostasis. The columns summarize mitochondrial functions in BECs (magenta), astrocytes (green), pericytes (purple), microglia (blue), and neurons (orange). Oxidative phosphorylation (OXPHOS), adenosine triphosphate (ATP) production, controlled reactive oxygen species (ROS) signaling, calcium (Ca^2+^) handling, metabolic flexibility, and mitochondrial quality control converge on tight-junction maintenance, selective transport, cerebral blood-flow regulation, immune surveillance, and neurovascular coupling. Ang-1, angiopoietin-1; BBB, blood-brain barrier; ECM, extracellular matrix; FAO, fatty-acid oxidation; NVU, neurovascular unit; TGF-β, transforming growth factor beta. Solid downward arrows indicate proposed direction of functional influence; double-headed horizontal arrows indicate reciprocal coupling among ATP production, ROS signaling, and redox/metabolic balance. No inhibition symbol is used. Pale background colors group processes by NVU cell type, whereas dark purple boxes denote the overarching input and homeostatic outcome. Flow diagram with five color-coded columns showing mitochondrial functions in endothelial cells, astrocytes, pericytes, microglia, and neurons. Cell-specific processes converge on ATP production, controlled reactive oxygen species signaling, and redox/metabolic balance, which in turn support tight-junction integrity, selective transport, vascular tone, immune surveillance, and neurovascular coupling. Downward arrows indicate proposed functional influence, and double-headed arrows indicate reciprocal coupling.

Growing evidence indicates that mitochondria function as dynamic signaling platforms that integrate metabolism, redox balance, calcium fluxes, innate immunity, cell fate decisions and epigenetic regulation (Shao et al., 2025). Within the NVU, these functions are particularly relevant because endothelial cells, astrocytes, pericytes, microglia and neurons depend on tightly coordinated metabolic and inflammatory responses to preserve BBB integrity and neurovascular homeostasis (Wang Y. et al., 2023; Liao et al., 2025). These cell-specific homeostatic functions are summarized in Figure 2.

#### 3.1.1 Calcium homeostasis and redox signaling

Under physiological conditions, mitochondrial calcium uptake supports metabolic adaptation by coupling ATP production to cellular demand. Conversely, excessive calcium accumulation can induce mitochondrial permeability transition, membrane depolarization and activation of cell death pathways (Serrat et al., 2022; de Oliveira, 2026). A failure in mitochondrial calcium sequestration causes a cytosolic overload that activates calcium-dependent proteases, which degrade binding proteins such as occludin (Jones et al., 2021; Song et al., 2024).

Although ROS are often viewed as harmful byproducts of respiration, low and tightly controlled ROS levels participate in physiological signaling pathways involved in angiogenesis, inflammatory responses and vascular adaptation. However, persistent mitochondrial oxidative stress promotes endothelial dysfunction, oxidation of tight junction proteins, activation of inflammatory transcription factors and BBB disruption. In BECs, mitochondrial ROS accumulation has been directly linked to vascular inflammation, transporter dysfunction and neurovascular uncoupling (Wang Y. et al., 2023; Wang et al., 2026).

#### 3.1.2 Quality control and connectivity

Efficient mitophagy prevents accumulation of dysfunctional mitochondria, excessive ROS production and release of pro-inflammatory mitochondrial components (Büttiker et al., 2023; Song et al., 2024). Recently, impaired mitophagy has emerged as a candidate mechanism that may connect chronic inflammation, metabolic dysfunction and neurovascular injury (Motta et al., 2023; Yusuf et al., 2025). Furthermore, as part of mitochondrial dynamics, fusion proteins such as mitofusin 1 (MFN1), mitofusin 2 (MFN2), and optic atrophy 1 (OPA1) promote mitochondrial connectivity and metabolic complementation, whereas fission proteins such as dynamin-related protein 1 (DRP1) facilitate mitochondrial segregation. Consequently, balanced mitochondrial dynamics are essential for cellular adaptation, stress resistance and distribution of mitochondria toward regions of high metabolic demand (Motta et al., 2023).

#### 3.1.3 Metabolism and epigenetic regulation

Finally, mitochondria are metabolic centers that processes lipids and amino acids, producing intermediates of the tricarboxylic acid (TCA) cycle that serve not only as fuel, but also as cofactors for epigenetic regulation (Kim et al., 2019; Song et al., 2024). Metabolites such as acetyl-CoA, α-ketoglutarate, succinate and fumarate regulate histone acetylation, DNA methylation and chromatin remodeling by serving as cofactors or inhibitors of epigenetic enzymes. Through this mechanism, mitochondrial dysfunction can reprogram gene expression in the endothelium, silencing protective barrier genes and perpetuating a long-term neuroinflammatory phenotype (Zong et al., 2024; Mihaylova et al., 2026).

### 3.2 Endothelial mitochondria and barrier function

Although BECs rely substantially on aerobic glycolysis, their exceptionally high mitochondrial density serves a specialized role as a regulatory hub for redox signaling and inflammatory gating, both essential for maintaining BBB function (Kim et al., 2022). Furthermore, mitochondrial integrity remains critical because mitochondrial stress in cerebrovascular endothelial cells has been shown to directly increase BBB permeability (Doll et al., 2015; Kim et al., 2022). Recent research using human induced pluripotent stem cells (iPSC)-derived models suggest that mitochondrial functionality, beyond ATP availability alone, influences the endothelial response to neuroinflammatory stress (Weber et al., 2022).

#### 3.2.1 Mitochondrial ROS and tight junction destabilization

Hypoxia–reoxygenation can increase ROS generation in BECs and compromise BBB integrity, supporting the idea that oxidative stress can translate mitochondrial or metabolic stress into tight junction instability (Schreibelt et al., 2007; Zehendner et al., 2013). High-resolution imaging in BEC cultures has demonstrated that an acute mitochondrial crisis, characterized by excessive ROS production, precedes the physical loss of barrier function (Doll et al., 2015). This mitochondrial ROS surge triggers the phosphorylation and subsequent degradation of claudin-5 and occludin (Doll et al., 2015; Wang Y. et al., 2023). In SZ-derived endothelial organoids, these mitochondrial deficits are intrinsically linked to increased paracellular permeability even in the absence of external inflammatory stimuli (Stankovic et al., 2024).

#### 3.2.2 Nuclear factor kappa B (NF-κB) activation and leukocyte adhesion

Recent transcriptomic analysis of the NVU under chronic social stress reveals a distinct reprogramming of vascular cells toward a pro-inflammatory state (Samuels et al., 2023). Mitochondrial fragmentation, often mediated by the DRP1 protein, acts as a critical signal that activates NF-κB, leading to the surface expression of adhesion molecules such as ICAM-1 and VCAM-1 (Haileselassie et al., 2020; Samuels et al., 2023). This process transforms the BECs from a restrictive barrier into an active docking site for peripheral leukocytes, a key step in the progression of neuroinflammatory psychiatric phenotypes (De Schepper et al., 2023; Samuels et al., 2023). In a mouse stroke model, lipopolysaccharide (LPS) aggravated BBB opening and was associated with impaired endothelial oxidative phosphorylation (OXPHOS), supporting a link between mitochondrial crisis, inflammation and barrier failure (Doll et al., 2015). *In vitro*, inflammatory stimulation of BECs increases VCAM-1 expression and monocyte adhesion through NF-κB/MAPK-dependent mechanisms (Lee et al., 2017), while silencing polymerase δ-interacting protein 2 (Poldip2) reduces TNF-induced VCAM-1 upregulation and leukocyte adhesion (Eidson et al., 2021).

#### 3.2.3 Oxidative damage and transporter dysfunction

Some studies mapping the metabolic plasticity of iPSC-derived BECs show that mitochondrial dysfunction impairs the activity of essential glucose and drug transporters (Weber et al., 2022). Furthermore, it has been observed that experimental hypoxia in brain capillary endothelial monolayers modifies expression of transporters such as GLUT1, P-gp, LAT1 and transferrin receptor while preserving barrier integrity, suggesting that transporter regulation may change before overt structural breakdown (Ozgür et al., 2022). In ischemic models, P-gp has also been implicated in BBB dysfunction through effects on endothelial autophagy, indicating that transporter changes are part of a broader stress-response program rather than passive consequences of barrier leakage (Huang et al., 2022).

### 3.3 Astrocytic mitochondria and vascular support

#### 3.3.1 Mitochondrial transfer to the endothelium

Astrocytes appear to be central regulators of the NVU, as they physically contact cerebral vessels through their endfeet and provide metabolic, redox and trophic support to endothelial cells. In this context, astrocytic mitochondria are not only required for astrocyte survival, but also for sustaining the functions that allow astrocytes to stabilize the BBB (Velmurugan et al., 2024).

In models of neurovascular distress, it has been observed that a subset of dentin matrix acidic phosphoprotein 1 (DMP1)-expressing astrocytes utilize a cluster of differentiation 38/cyclic ADP-ribose (CD38/cADPR)-dependent pathway to donate mitochondria to damaged endothelial cells through astrocytic endfeet, a process that is vital for repairing the barrier and restoring its restrictive function; reducing this mitochondrial transfer impaired BBB integrity and increased barrier leakage (Liu et al., 2024). A related *in vivo* study also demonstrated astrocyte-to-endothelial and astrocyte-to-pericyte mitochondrial transfer in brain capillaries (Velmurugan et al., 2024), suggesting that mitochondrial exchange may be an underrecognized form of neurovascular support. This astrocytic-to-endothelial transfer is regulated by the fusion protein MFN2, whereas experimental depletion of astrocytic MFN2 results in vascular leakage and impairs the ability of the NVU to recover from inflammatory insults (Liu et al., 2024).

#### 3.3.2 Metabolic coupling and neurovascular synchronization

Astrocytes regulate glucose utilization, glycogen storage, glutamate handling, and lactate production, allowing them to adapt local energy supply to neuronal and vascular demand. Although much of the classical lactate literature is not BBB-specific, it supports the broader concept that astrocytic metabolism links neurotransmitter clearance, energetic support, and perivascular homeostasis. Human iPSC-derived BBB models further suggest that mitochondrial enzyme signatures within NVU cells influence glucose metabolism and nutrient transporter maintenance (Weber et al., 2022). Thus, astrocytic mitochondrial dysfunction may weaken glutamate buffering, disturb metabolic coupling, and increase excitotoxic or inflammatory stress near the BBB.

#### 3.3.3 Antioxidant defense and structural stability

Astrocytic mitochondria function as the primary antioxidant shield of the NVU. In astrocyte–endothelial co-culture systems, Huang S. F. et al. (2020) showed that astrocyte-derived glutathione supports endothelial barrier stability, preserves tight junction organization and protects endothelial cells from injury-induced barrier dysfunction. In this context, astrocytes further maintain BBB homeostasis through trophic signaling. Sonic Hedgehog released by astrocytes promotes endothelial tight junction expression and barrier integrity, whereas IL-1β-induced suppression of astrocytic Sonic Hedgehog increases BBB permeability and downregulates endothelial tight junction proteins (Wang et al., 2014). Similarly, astrocyte-derived TGF-β1 has been shown to enhance endothelial barrier function by activating Hedgehog signaling and consolidate tight junctions. These studies indicate that astrocytes stabilize the BBB not only through physical contact, but also through secreted signals that regulate endothelial phenotype (Fu et al., 2021).

### 3.4 Pericyte mitochondria and capillary stability

#### 3.4.1 Vascular stability and reprogramming in response to stress

Pericytes are indispensable regulators of microvascular stability within the NVU. Experimental studies show that pericyte loss destabilizes the BBB, increases vascular permeability, and alters cerebral blood flow regulation (Nikolakopoulou et al., 2017; Sun et al., 2021). Chronic hypoperfusion and stress-related models further suggest that pericyte detachment and vascular transcriptional reprogramming can precede or accompany BBB vulnerability and immune activation (Liu Q. et al., 2019; Samuels et al., 2023). *In vivo* imaging studies demonstrated that capillary pericytes can contract and modulate local cerebral blood flow in response to neuronal activity, indicating that these cells participate directly in matching metabolic demand with microvascular perfusion (Liu Q. et al., 2019). Because these processes require dynamic cytoskeletal remodeling, calcium signaling and oxidative balance, mitochondrial homeostasis is likely essential for preserving pericyte contractility and vascular responsiveness. Experimental hypoxic and ischemic conditions impair pericyte mitochondrial membrane potential, increase mitochondrial ROS production and promote pericyte dysfunction, linking metabolic stress to microvascular instability (Sun et al., 2021).

Increasing evidence indicates that pericyte dysfunction or metabolic impairment contributes directly to chronic neuroinflammatory states, partly through the regulation of leukocyte adhesion molecules in endothelial cells (Zhang et al., 2023).

#### 3.4.2 Mitochondrial dynamics and permeability

Mitochondrial dysfunction mediated by the fission protein DRP1 has been identified as a key mechanism propagating BBB deterioration (Haileselassie et al., 2020). Excessive fragmentation of mitochondria in the vascular compartment not only reduces energy efficiency but also acts as a pro-inflammatory signal that increases paracellular permeability (Haileselassie et al., 2020). Under conditions of severe oxidative stress, this damage can progress to mitophagy-dependent ferroptosis, a regulated cell death process that destroys the capillary structure and degrades the barrier (Li et al., 2022).

#### 3.4.3 ECM and perivascular signaling

As mentioned above, perivascular cells can induce phagocytic states in microglia via the secreted phosphoprotein 1 (SPP1) pathway, resulting in synapse engulfment and ECM remodeling (De Schepper et al., 2023). This change in perivascular cell function, driven by metabolic stress signals, alters basement membrane composition and trophic signaling, compromising normal angiogenesis and long-term stability of the capillary network (De Schepper et al., 2023; Samuels et al., 2023). Pericytes also contribute to ECM organization and vascular basement membrane maintenance. They regulate deposition and remodeling of laminin, collagen IV and fibronectin, thereby stabilizing endothelial adhesion and vascular architecture (Nikolakopoulou et al., 2017). Experimental depletion of pericytes disrupts basement membrane integrity and facilitates endothelial hyperpermeability (Sun et al., 2021). Importantly, oxidative and mitochondrial stress can activate matrix-remodeling pathways and alter pericyte–endothelial communication, suggesting that mitochondrial dysfunction may indirectly destabilize the ECM and amplify BBB disruption (Nikolakopoulou et al., 2017; Sun et al., 2021).

### 3.5 Microglial mitochondria and perivascular inflammation

#### 3.5.1 Immunometabolic reprogramming, mitochondrial stress and BBB injury

Microglia are metabolically plastic cells whose inflammatory behavior is closely linked to mitochondrial function. Their ability to transition between a state of homeostatic surveillance and one of pro-inflammatory activation depends on a metabolic reprogramming called immunometabolic switching, where the cell prioritizes aerobic glycolysis to sustain rapid defensive functions (Samuels et al., 2023). This metabolic reprogramming has been shown experimentally in amyloid-β–exposed microglia, where acute activation was accompanied by a transition from OXPHOS toward glycolysis, while failure to sustain this metabolic adaptation contributed to impaired microglial function (Baik et al., 2019). Similar work in LPS-stimulated microglia showed increased glycolysis together with mitochondrial respiratory dysfunction (Sangineto et al., 2023).

#### 3.5.2 The SPP1+ phagocytic state and structural degradation

This immunometabolic shift is relevant for BBB homeostasis because activated microglia can localize near cerebral vessels and directly influence barrier permeability. In a systemic inflammation model, Haruwaka et al. (2019) showed that vessel-associated microglia initially helped preserve BBB integrity through claudin-5-related mechanisms and physical contact with endothelial cells. However, during sustained inflammation, microglia changed behavior, phagocytosed astrocytic endfeet and contributed to increased BBB permeability (Haruwaka et al., 2019). This suggests that microglia can have dual context-dependent effects on the BBB: protective during early inflammatory responses but damaging when activation is prolonged. This phenotypic change, driven by signals from the vascular microenvironment, allows microglia to phagocytize astrocytic endfeet and synaptic elements, eliminating the life support that the astrocyte provides to the endothelium (De Schepper et al., 2023). These findings suggest that metabolically activated microglia can contribute to barrier vulnerability before overt structural breakdown, although this sequence requires further validation in psychiatric models.

#### 3.5.3 Mitochondrial ROS and inflammasome activation

Mitochondrial ROS represent one of the main links between microglial metabolic stress and inflammatory amplification. In experimental models, mitochondrial impairment in microglia enhances NLRP3 inflammasome activation, increases IL-1β–associated inflammatory signaling and worsens neuroinflammatory injury (Sarkar et al., 2017). Wang Y. et al. (2023) demonstrated this in microglial cell culture and animal models of Parkinsonian neurodegeneration, while additional toxicological models showed that mitochondrial ROS and altered mitophagy can drive NLRP3 activation in BV2 microglial cells. However, mitophagy limits mitochondrial ROS production and prevents the release of mitochondrial danger signals capable of sustaining inflammasome activation (Zhong et al., 2016). Experimental studies have shown that defective mitochondrial clearance promotes NLRP3-dependent inflammation, whereas enhancement of Parkin-dependent mitophagy can reduce neuroinflammatory responses in models of intermittent hypoxia (Wu et al., 2021). These findings suggest that impaired microglial mitophagy may favor a self-perpetuating loop: damaged mitochondria accumulate, mitochondrial ROS increases, NLRP3 becomes activated, and inflammatory mediators further damage the neurovascular environment.

## 4 Mechanisms linking mitochondrial dysfunction to BBB disruption

BBB disruption is a dynamic process shaped by intracellular signaling, inflammation, metabolic stress, and interactions among NVU cells (Shao et al., 2025; Wang et al., 2025). In BECs, these stimuli can remodel the cytoskeleton, transporters, junctions, and transcellular or paracellular permeability (Brailoiu et al., 2024). Mitochondria integrate redox signaling, calcium homeostasis, ATP production, metabolite availability, quality control, apoptosis, and innate immunity (Luo et al., 2024; Shao et al., 2025). In rat BECs, excessive mitochondrial calcium and ROS accompanied junctional disruption, cytoskeletal remodeling, reduced monolayer resistance, and greater *in vivo* leakage (Brailoiu et al., 2024). Other endothelial and neurovascular-injury models link impaired respiration, excessive ROS, defective mitophagy, and membrane damage with inflammatory signaling, apoptosis, and altered junctional proteins; restoring mitochondrial quality control can partly preserve barrier properties (Lu et al., 2025; Shao et al., 2025; Zhao et al., 2025). Metabolic reprogramming may likewise influence whether endothelial cells retain a quiescent barrier-supportive phenotype or become activated and permeable (Wang et al., 2025).

Importantly, mitochondrial contributions to barrier dysfunction are not restricted to endothelial cells. Altered mitochondrial function in astrocytes, pericytes, and microglia can modify antioxidant support, calcium-dependent intercellular communication, inflammatory mediator release, ECM maintenance, and endothelial survival, thereby amplifying barrier disruption through multicellular NVU mechanisms (Qin et al., 2024; Shao et al., 2025). These interactions are likely to be context dependent, because mitochondrial ROS can function as physiological second messengers at moderate levels but promote oxidative damage, inflammasome activation, cytoskeletal contraction, and cell death when production becomes excessive or mitochondrial clearance is impaired (Luo et al., 2024; Qin et al., 2024). Accordingly, mitochondrial dysfunction should not be interpreted as a single linear pathway, but as a nodal disturbance capable of simultaneously affecting endothelial junctions, inflammatory signaling, metabolic adaptation, and communication among NVU cells.

In psychiatric disorders, however, the strength of evidence varies considerably across levels of analysis. Clinical and postmortem studies support alterations in BBB-associated markers and provide evidence of mitochondrial, oxidative, and inflammatory abnormalities in subsets of patients with SZ, BD, and MDD, but these findings do not yet demonstrate that mitochondrial dysfunction within BECs is a primary cause of BBB leakage in most psychiatric populations (Wakonigg Alonso et al., 2024; Zhang et al., 2025). Experimental brain injury models have linked BBB-regulatory molecular pathways to SZ-like and anxiety-related behaviors, although such models do not reproduce the multifactorial pathophysiology of primary psychiatric disorders (Lin et al., 2024). Therefore, the following sections distinguish mechanisms demonstrated directly in BECs, multicellular BBB systems, or *in vivo* barrier models from mechanisms that remain biologically plausible but require validation in patient-derived NVU models, longitudinal clinical cohorts, and psychiatric populations stratified by inflammatory, metabolic, or mitochondrial phenotypes.

### 4.1 Mitochondrial ROS and tight junction remodeling

Mitochondrial dysfunction may increase ROS and remodel endothelial junctions. In brain endothelial models, ROS activate Ras homolog family member A, phosphoinositide 3-kinase, and protein kinase B signaling, promoting cytoskeletal rearrangement, claudin-5 and occludin redistribution, and monocyte migration (Schreibelt et al., 2007). Experimental endothelial and ischemic studies also associate oxidative stress with increased permeability, although comparable effects in psychiatric patients remain unestablished (Lochhead et al., 2010). Tempol attenuated oxidative-stress-associated occludin remodeling and leakage. After focal cerebral ischemia, permeability changes paralleled reduced claudin-5, occludin, and ZO-1 expression and redistribution, indicating coordinated junctional remodeling rather than alteration of one protein (Jiao et al., 2011).

More directly linking mitochondrial dysfunction to BBB permeability, Doll et al. (2015) showed that LPS reduced oxidative phosphorylation and respiratory chain complex expression in cerebrovascular endothelial cells. Pharmacological inhibition of mitochondrial function with rotenone, carbonyl cyanide p-trifluoromethoxyphenylhydrazone (FCCP) or oligomycin increased BBB permeability *in vitro*, disrupted ZO-1 staining and produced gaps between endothelial cells. *In vivo*, rotenone worsened BBB leakage and stroke outcome after transient middle cerebral artery occlusion (Doll et al., 2015). These data support the idea that mitochondrial impairment can directly destabilize endothelial barrier properties in experimental BBB models.

### 4.2 Mitochondrial control of endothelial inflammation

In experimental systems, mitochondrial and oxidative stress can shift BECs toward a pro-inflammatory phenotype characterized by NF-κB activation and increased adhesion-molecule expression. True et al. (2000) showed that H_2_O_2_ and tumor necrosis factor alpha (TNF-α) regulate NF-κB activation and ICAM-1 transcription in human endothelial cells, while Kim et al. (2008) demonstrated that visfatin increased ICAM-1 and VCAM-1 expression through ROS-dependent NF-κB activation. As mentioned above, inflammasome activation represents another route by which mitochondrial dysfunction can amplify neurovascular inflammation. In this regard, Gong et al. (2018) showed in cerebral ischemia/reperfusion models that NLRP3 inflammasome activation appeared first in microglia and later in neurons and microvascular endothelial cells; importantly, mitochondrial dysfunction contributed to NLRP3 activation, and mitochondrial protection reduced NLRP3, apoptosis-associated speck-like protein containing a CARD (ASC), cleaved caspase-1, IL-1β and IL-18. These findings support the possibility that mitochondrial stress may amplify inflammatory signaling across neurovascular cell types during experimentally induced BBB injury.

Other studies further demonstrated that NF-κB–dependent transcriptional programs associated with increased expression of adhesion molecules and pro-inflammatory cytokines facilitate the recruitment and adhesion of peripheral leukocytes and allow their infiltration into the CNS (Doll et al., 2015; Haileselassie et al., 2020; Samuels et al., 2023). In the same way, inflammatory stimulation with TNF-α or LPS further enhances mitochondrial ROS production, creating a feed-forward loop in which oxidative stress and inflammatory activation reinforce each other, while silencing of mitochondrial-associated regulators such as Poldip2 reduces VCAM-1 expression and leukocyte adhesion, supporting a direct role for mitochondrial signaling in endothelial immune activation (Eidson et al., 2021).

Mitochondrial damage can also initiate innate immune signaling through the release of mitochondrial DNA (mtDNA). In retinal microvascular endothelial cells, Guo et al. (2020) demonstrated that pathological stimuli induced mitochondrial damage, cytosolic mtDNA leakage and activation of cyclic GMP–AMP synthase–stimulator of interferon genes (cGAS–STING) signaling, driving non-infectious inflammatory activation. Similarly, Huang L. S. et al. (2020) showed that endothelial mtDNA activates cGAS signaling and generates cGAMP, linking mitochondrial injury to endothelial inflammatory and repair responses. Finally, transcriptomic studies in contexts of social defeat stress confirm that this mitochondria-driven genetic reprogramming facilitates the recruitment and adhesion of peripheral leukocytes, allowing their infiltration into the CNS (Samuels et al., 2023).

### 4.3 Calcium dysregulation and endothelial permeability

Calcium dysregulation can increase BBB permeability through junctional and cytoskeletal mechanisms. Calcium modulation alters adherens- and tight-junction function in brain microvessel endothelial cells (Brown and Davis, 2002), while calcium overload activates myosin light-chain kinase (MLCK), endothelial contraction, and VE-cadherin destabilization (Haileselassie et al., 2020; Wang Y. et al., 2023). Excessive dynamin-related protein 1-mediated mitochondrial fragmentation may intensify this process. In hypoxic models, myosin light-chain inhibition prevented barrier disruption (Kuhlmann et al., 2007), and trifluoperazine reduced post-ischemic permeability by suppressing MLCK/phosphorylated myosin light-chain signaling and actin contraction (Zhang W. et al., 2024). These findings identify calcium-dependent contractility as a modifiable experimental mechanism, not yet a demonstrated psychiatric pathway.

### 4.4 Impaired mitophagy and barrier vulnerability

Efficient mitophagy, mediated by the PTEN-induced kinase 1 (PINK1) and Parkin proteins, is vital for preserving energy metabolism and coupling the mitochondrial network (Quinn et al., 2020). Impairment of this pathway significantly contributes to neuroinflammation and oxidative stress, factors that exacerbate endothelial dysfunction (Lou et al., 2020; Quinn et al., 2020). Nakahira et al. (2011) showed that loss of autophagy proteins promoted accumulation of damaged mitochondria, increased mitochondrial ROS and enhanced release of mitochondrial DNA, thereby amplifying NLRP3 inflammasome-dependent inflammation. In endothelial cells, mitophagy appears to function as a protective response against metabolic stress. Wu et al. (2015) showed that the PINK1–Parkin pathway is activated under metabolic stress and that induction of mitophagy protects mitochondrial integrity and prevents metabolic stress-induced endothelial injury.

In BECs, LPS-induced injury involves mitochondrial apoptosis, Omi/HtrA2 translocation, X-linked inhibitor of apoptosis protein inhibition, and loss of mitochondrial membrane potential (Hu et al., 2019). In neurotoxic-protein models, cluster of differentiation 36/PINK1/Parkin signaling can induce mitophagy-associated ferroptosis, pericyte loss, and vascular damage (Li et al., 2022). Under metabolic or ischemic stress, dysregulated autophagy may also promote occludin degradation; occludin colocalization with autophagosomes supports direct junctional turnover before complete cell death (Kim et al., 2020). Thus, mitochondrial quality-control failure may progress from reversible stress to endothelial or pericyte loss and barrier instability.

### 4.5 mtDNA as a danger signal

Defective autophagy promotes mitochondrial damage, mtDNA release, and NLRP3 activation, while oxidized mtDNA can directly activate NLRP3 during apoptosis (Nakahira et al., 2011; Shimada et al., 2012). In retinal microvascular endothelial cells, mitochondrial permeability-transition pore opening permits cytosolic mtDNA escape and cGAS–STING activation, increasing interferon beta, NF-κB phosphorylation, and ICAM-1 (Guo et al., 2020; Huang L. S. et al., 2020). Extracellular mtDNA can also engage Toll-like receptor 9 and promote adhesion-molecule expression and leukocyte recruitment (Gambardella et al., 2019; de Oliveira et al., 2021; Jauhari et al., 2026). Human evidence is less direct: cerebrospinal-fluid mtDNA has been associated with viral burden, inflammation, and neurocognitive impairment in HIV, and cell-free mtDNA is measurable after acute brain injury (Mehta et al., 2017; Kayhanian et al., 2022). These findings establish mtDNA as an inflammatory signal but not as a proven mediator of psychiatric BBB dysfunction.

For psychiatric disorders, these pathways should be understood as biologically plausible but still underexplored: the inflammatory role of mtDNA is well-supported in immune, endothelial and CNS injury models, whereas direct evidence linking mtDNA-driven BBB disruption to psychiatric phenotypes remains limited. The relevance of this mechanism is reflected in clinical studies that associate mtDNA levels with psychopathological states. Circulating cf-mtDNA has been identified as significantly elevated in the plasma of patients with MDD, suggesting active mitochondrial release due to chronic cellular stress (Lindqvist et al., 2018). Furthermore, research in suicide attempt survivors has reported elevated levels of circulating cf-mtDNA associated with HPA axis hyperactivity, proposing mtDNA as a biomarker of tissue damage and extreme metabolic stress (Lindqvist et al., 2016).

### 4.6 Metabolic reprogramming of BBB cells

The BBB is a metabolically dynamic structure whose function depends on the coordinated activity of endothelial cells, astrocytes, pericytes and microglia. Lee et al. (2022) showed that endothelial-derived lactate is required for pericyte function and BBB maintenance. In fact, during physiological conditions, BECs rely predominantly on glycolysis despite their high mitochondrial content, whereas astrocytes, pericytes and microglia display considerable metabolic flexibility. However, under inflammatory or oxidative stress, neurovascular cells undergo metabolic reprogramming characterized by alterations in glycolysis, OXPHOS, oxidized/reduced nicotinamide adenine dinucleotide (NAD+/NADH) balance and lipid metabolism (Kim et al., 2022).

Mitochondrial complex I dysfunction, observed in disorders such as SZ and BD, alters the NAD+/NADH ratio, triggering a cascade of redox dysregulation (Dwir et al., 2019). Decreased mitochondrial NAD+ limits the activity of protective enzymes, exacerbating ROS damage and activating pro-inflammatory pathways such as the receptor for advanced glycation end products (RAGE), directly linking energy metabolism to clinical neuroinflammation (Dwir et al., 2019). Interestingly, it has been observed that during inflammatory activation due to chronic psychobiological stress, NVU cells undergo a metabolic transition similar to the Warburg effect, where the cell prioritizes aerobic glycolysis over oxidative phosphorylation by altering gene expression patterns, promoting glycolytic pathways to generate rapid energy for the immune response, which directly impacts the stability of the BBB (Samuels et al., 2023). In fact, when the energy demand of the inflammatory response exceeds mitochondrial capacity, a mitochondrial crisis occurs that drastically reduces ATP levels, causing the collapse of ion pumps and the opening of intercellular junctions (Doll et al., 2015).

Mitochondria also regulate the synthesis and degradation of complex lipids that form the endothelial membrane. Therefore, metabolic reprogramming that favors the accumulation of lipid peroxides, mediated by the CD36/PINK/Parkin pathway, can lead to ferroptosis of NVU components (Li et al., 2022). This failure in lipid metabolism not only destroys mitochondria but also physically degrades the barrier by inducing programmed cell death of pericytes and endothelial cells, eliminating the vital support of the microvasculature (Parodi-Rullán et al., 2019; Li et al., 2022). However, metabolic stress can also activate mitochondrial quality control pathways, as Wu et al. (2015) demonstrated that PINK1-Parkin-mediated mitophagy protects mitochondrial integrity and prevents endothelial damage induced by metabolic stress.

### 4.7 MMPs and ECM remodeling

The degradation of the ECM and junctional complexes is the final step in the transition from an intact to a permeable barrier (Liu et al., 2012). MMPs, specifically the gelatinases MMP-2 and MMP-9 (Feng et al., 2011), are the main effectors of this process, acting under the direct control of oxidative stress and inflammation of mitochondrial origin (Kim et al., 2003; Lehner et al., 2011). Feng et al. (2011) showed in an *in vitro* BBB model that leukemic cells secreted MMP-2 and MMP-9, impaired ZO-1, claudin-5 and occludin, and increased BBB permeability. The same study showed that MMPs inhibition or MMP-2/MMP-9 knockdown reduced tight junction disruption, supporting a causal role for these enzymes in BBB damage.

**FIGURE 3:**
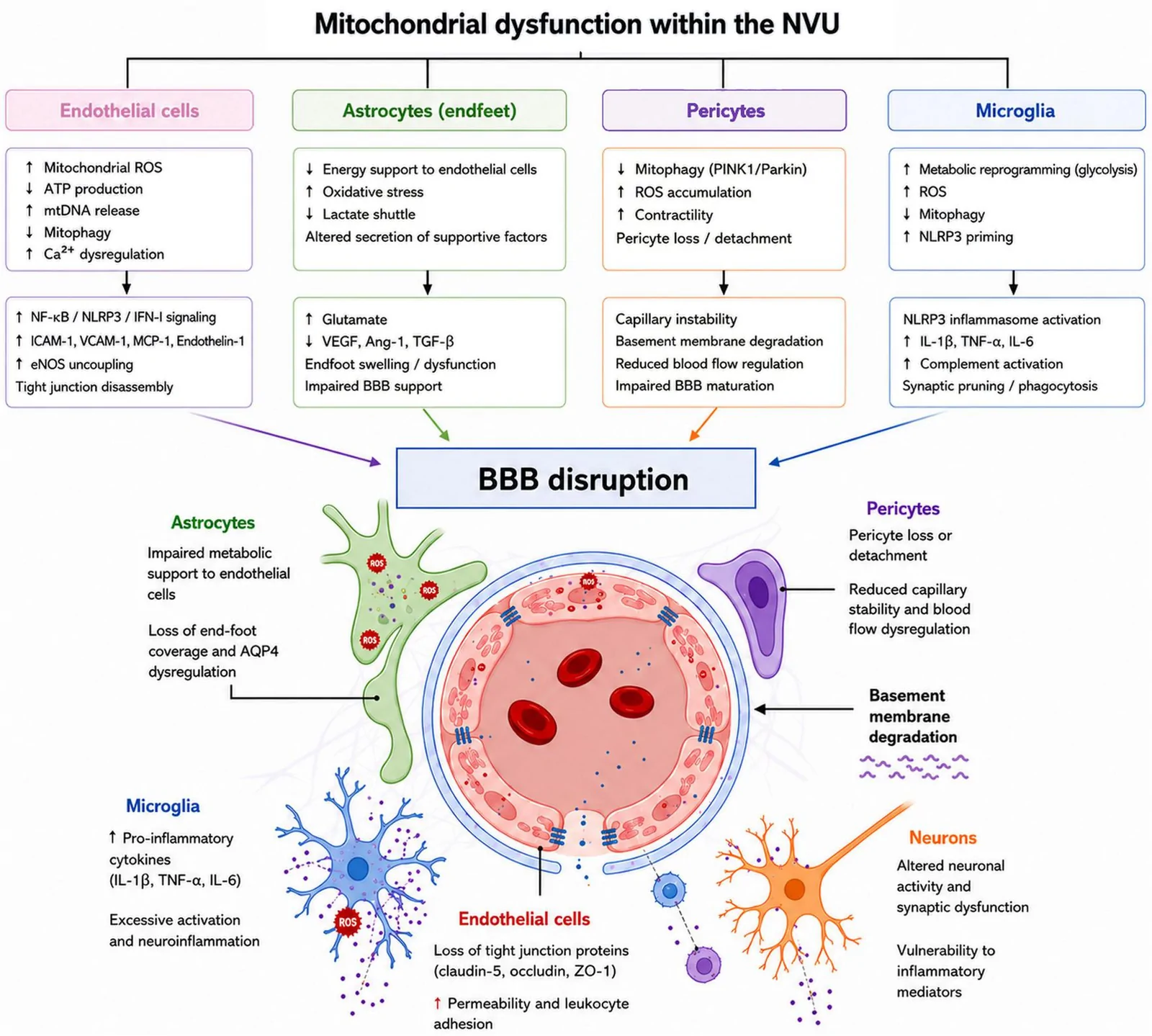
Proposed cell-specific pathways linking mitochondrial stress to BBB dysregulation. The upper panel summarizes candidate mechanisms in BECs (magenta), astrocytes (green), pericytes (purple), and microglia (blue); colored arrows converge on the central BBB-dysregulation node. The lower panel illustrates associated structural and functional changes in endothelial cells (red/pink), astrocytes (green), pericytes (purple), microglia (blue), and neurons (orange). In BECs, mitochondrial ROS and mitochondrial DNA (mtDNA) release may engage nuclear factor kappa B (NF-κB), NOD-, LRR- and pyrin domain-containing protein 3 (NLRP3), and type I interferon (IFN-I) signaling, with increased intercellular adhesion molecule 1 (ICAM-1), vascular cell adhesion molecule 1 (VCAM-1), and monocyte chemoattractant protein 1 (MCP-1), endothelial nitric oxide synthase (eNOS) uncoupling, and tight-junction instability. Astrocytic, pericytic, and microglial mitochondrial alterations may reduce trophic/metabolic support, capillary stability, and mitophagy while increasing inflammatory signaling. Ang-1, angiopoietin-1; AQP4, aquaporin-4; BBB, blood-brain barrier; IL, interleukin; NVU, neurovascular unit; PINK1, PTEN-induced kinase 1; ROS, reactive oxygen species; TGF-β, transforming growth factor beta; TNF-α, tumor necrosis factor alpha; VEGF, vascular endothelial growth factor; ZO-1, zonula occludens-1. Solid arrows indicate proposed direction of influence; ↑ and ↓ indicate relative increases and decreases, respectively. The Figure contains no separate inhibitory bar; decreased activity or abundance is indicated by ↓. These pathways synthesize experimental and associative human evidence and should not be interpreted as established causal sequences in psychiatric disorders. Two-level schematic of proposed cell-specific mitochondrial contributions to blood-brain barrier dysregulation. The upper level shows endothelial, astrocytic, pericytic, and microglial mitochondrial alterations converging on barrier dysfunction. The lower level shows associated tight-junction instability, greater permeability and leukocyte adhesion, basement-membrane remodeling, pericyte detachment, impaired astrocytic endfeet, microglial inflammation, and neuronal vulnerability. Up and down arrows indicate relative increases and decreases.

**FIGURE 4:**
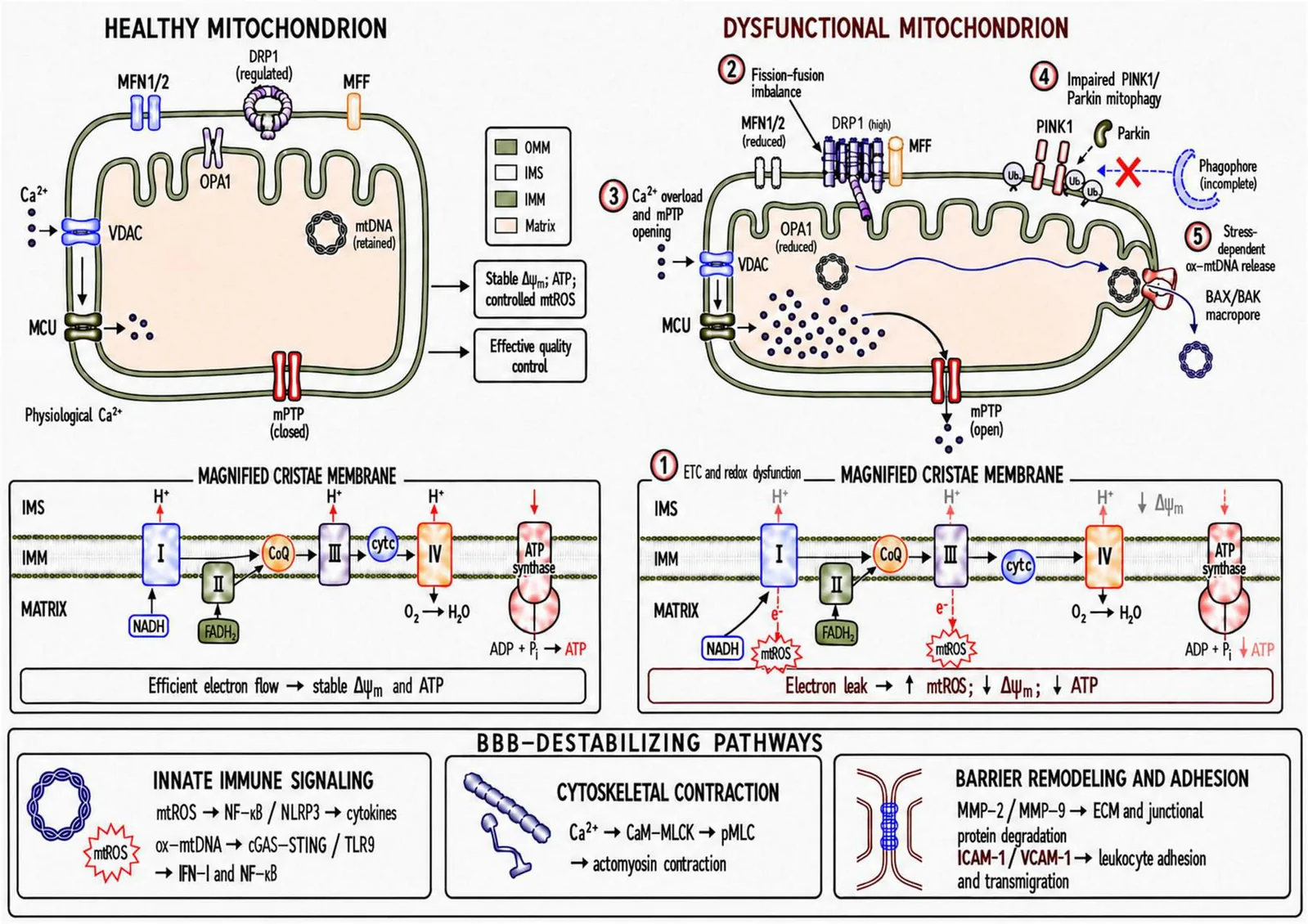
The healthy mitochondrion (left) illustrates balanced mitochondrial dynamics, physiological Ca^2 +^ handling, efficient oxidative phosphorylation (OXPHOS), and quality control. MFN1/2 and OPA1 support fusion and cristae organization, whereas regulated DRP1 recruitment through MFF supports fission. Ca^2 +^ enters sequentially from the cytosol through voltage-dependent anion channel (VDAC) in the outer mitochondrial membrane (OMM), traverses the intermembrane space (IMS), and reaches the matrix through the mitochondrial calcium uniporter (MCU) in the inner mitochondrial membrane (IMM); the mitochondrial permeability transition pore (mPTP) remains closed. The magnified cristae membrane shows the electron transport chain (ETC) within the IMM: electrons from reduced nicotinamide adenine dinucleotide (NADH) enter complex I, electrons from reduced flavin adenine dinucleotide (FADH_2_) enter complex II, and both converge at coenzyme Q (CoQ), followed by complex III, cytochrome c, and complex IV, where O_2_ is reduced to H_2_O. Complexes I, III, and IV translocate H^+^ from the matrix to the IMS; H^+^ return through ATP synthase supports ATP synthesis on the matrix side. The dysfunctional mitochondrion (right) depicts ETC and redox dysfunction, fission-fusion imbalance, impaired PINK1/Parkin-dependent mitophagy, Ca^2 +^ overload with mPTP opening, and stress-dependent oxidized mitochondrial DNA (ox-mtDNA) release through BCL2-associated X protein (BAX)/BCL2 antagonist/killer 1 (BAK) macropores. Electron leakage at complexes I and III increases mitochondrial reactive oxygen species (mtROS) generation and is associated with reduced mitochondrial membrane potential (ΔΨm) and ATP production. Increased DRP1–MFF activity and reduced MFN1/2 and OPA1 are associated with fragmentation and cristae disorganization. Excess matrix Ca^2 +^ favors mPTP opening and outward ion/solute flux. Dysfunctional PINK1/Parkin signaling and incomplete phagophore recruitment limit mitophagic progression and clearance. These mitochondrial danger signals converge on blood-brain barrier (BBB)-destabilizing pathways, including mtROS-driven nuclear factor kappa B (NF-κB)/NLR family pyrin domain-containing 3 (NLRP3) signaling, ox-mtDNA-mediated cyclic GMP–AMP synthase (cGAS)–stimulator of interferon genes (STING)/Toll-like receptor 9 (TLR9) signaling, Ca^2 +^–calmodulin (CaM)–myosin light-chain kinase (MLCK)–phosphorylated myosin light chain (pMLC)-dependent actomyosin contraction, and matrix metalloproteinase 2/9 (MMP-2/9)- and intercellular/vascular cell adhesion molecule 1 (ICAM-1/VCAM-1)-associated barrier remodeling and leukocyte transmigration. Black arrows indicate the direction of electron, ion, molecular, or signaling flow; red dashed arrows indicate pathological electron leakage from ETC complexes I and III; red upward arrows indicate H^+^ translocation from the matrix to the IMS; and downward arrows indicate reduced ΔΨm or ATP output. The red X denotes impaired phagophore recruitment/mitophagic progression. Color coding identifies mitochondrial compartments: OMM, pale green; IMS, white; IMM/cristae, green; and matrix, pale beige. Healthy-state annotations are green, dysfunctional-state annotations are dark red, mtROS is shown in red, and mtDNA/ox-mtDNA is shown in dark blue. Side-by-side schematic comparing healthy and dysfunctional mitochondria, with enlarged inner mitochondrial membrane cristae below each organelle. The healthy mitochondrion shows balanced fission–fusion proteins, calcium entry through outer- and inner-membrane channels into the matrix, closed permeability transition pore, retained mitochondrial DNA, and efficient electron transport from NADH and FADH_2_ to oxygen through complexes I–IV and ATP synthase. The dysfunctional mitochondrion shows increased fission, reduced fusion proteins, calcium accumulation in the matrix, pore opening, electron leakage from complexes I and III into the matrix with increased mitochondrial reactive oxygen species, lower membrane potential and ATP production, impaired PINK1/Parkin mitophagy, and oxidized mitochondrial DNA release through BAX/BAK macropores. The lower panel links these signals to inflammatory signaling, actomyosin contraction, barrier remodeling, and leukocyte adhesion pathways that destabilize the blood-brain barrier.

This MMP-mediated occludin cleavage may further worsen BBB injury by producing fragments that promote endothelial cell death after ischemic stroke (Pan et al., 2021). Interestingly, cytokine-driven pathways can converge on this same mechanism, as McMillin et al. (2015) showed that TGF-β1 increased BBB permeability through SMAD3-dependent MMP-9 upregulation and claudin-5 downregulation. Together, these studies support a model in which MMP-2 and MMP-9 translate inflammatory, hypoxic and ischemic stress into structural BBB remodeling. The mitochondrial mechanisms that drive BBB disruption are summarized in Figure 3. Complementary molecular-level pathways are detailed in Figure 4.

## 5 The mitochondrial–BBB axis across neuroinflammatory psychiatric disorders

The mitochondrial–BBB axis is best viewed as a convergent, potentially bidirectional framework rather than a universal causal pathway. SZ, MDD, BD, ASD, and PTSD show partially overlapping immune, redox, mitochondrial, and neurovascular abnormalities, but the strength and type of evidence differ substantially across disorders (Konstantinou and Warsh, 2026).

### 5.1 Strength of evidence in SZ

Among the disorders considered in this review, SZ currently has some of the most direct human evidence of BBB and neurovascular abnormalities. Postmortem studies have reported reduced hippocampal claudin-5 expression and alterations in tight-junction-related transcripts and proteins, including claudin-5, claudin-12, and ZO-1. Some of these changes were associated with illness duration and age at onset across major psychiatric disorders (Greene et al., 2020). Ultrastructural investigations have additionally described vacuolar degeneration and other abnormalities affecting cerebral endothelial cells and astrocytic endfeet in individuals with SZ (Najjar et al., 2013, 2017). These findings provide tissue-level evidence of altered BBB-associated biology, although postmortem designs cannot determine whether the abnormalities preceded illness onset or developed secondary to chronic disease, medication exposure, cardiometabolic comorbidity, agonal conditions, or tissue-processing factors.

Recent neuroimaging provides complementary *in vivo* evidence. Moussiopoulou et al. (2025) used dynamic contrast-enhanced magnetic resonance imaging (DCE-MRI) to compare 41 individuals with SZ-spectrum disorders and 40 healthy controls. The patient group showed a widespread pattern of higher contrast-agent leakage across several brain regions implicated in SZ. However, the estimated permeability changes were not significantly associated with cognition, symptom severity, peripheral inflammatory markers, or the CSF/serum albumin quotient. These findings indicate that subtle BBB abnormalities can be detected in living patients but do not demonstrate that barrier leakage causes psychiatric symptoms or cognitive dysfunction. Longitudinal studies are required to establish whether these abnormalities precede, accompany, or follow clinical exacerbation.

Human peripheral and central biomarker studies also support the coexistence of inflammatory, redox, bioenergetic, and vascular abnormalities. Cross-disorder and SZ-specific studies have reported altered peripheral cytokine networks, soluble adhesion molecules, S100B, CSF/serum albumin quotient (QAlb), lactate, glutathione, oxidative-stress markers, and circulating mitochondrial DNA (Yuan et al., 2019; Meixensberger et al., 2020; Murray et al., 2024). Studies in first-episode and drug-naïve patients further indicate that redox abnormalities can be detected early and are not necessarily explained exclusively by prolonged antipsychotic exposure. Nevertheless, these measures are not specific to BECs and may be influenced by smoking, adiposity, infection, medication, platelet or leukocyte composition, and systemic vascular disease.

Patient-derived cellular models provide a complementary mechanistic level of evidence. BECs and organoids derived from individuals with SZ have shown increased permeability, altered angiogenic properties, and abnormalities in pathways related to endothelial metabolism and mitochondrial function (Stankovic et al., 2024). Although these models retain aspects of the participants’ genetic background, they cannot reproduce cerebral blood flow, immune-cell trafficking, systemic metabolism, or the complete cellular organization of the NVU. They should therefore be interpreted as evidence of cell-autonomous endothelial vulnerability rather than direct demonstrations of *in vivo* BBB disruption.

Additional evidence comes from 22q11.2 deletion syndrome, a genetic condition associated with markedly elevated psychosis risk. Crockett et al. (2025) identified mitochondrial abnormalities in iPSC-derived BECs obtained from individuals with 22q11.2 deletion syndrome. Bezafibrate improved mitochondrial and barrier-related functions in these cells, while parallel effects on BBB integrity and social-memory performance were observed in a mouse model. Because the human findings were obtained from a specific genetic syndrome and the behavioral effects were demonstrated in mice, these results provide mechanistic plausibility but cannot be generalized to all individuals with SZ.

Human studies indicate that inflammatory, oxidative, mitochondrial, endothelial, and BBB-related abnormalities coexist in subsets of individuals with SZ, while experimental models provide plausible links among mitochondrial stress, junctional organization, and neurovascular signaling. Their temporal order remains unresolved; the axis should therefore be considered a candidate framework for an inflammatory or neurovascular subgroup, not an established cause of SZ or its symptoms.

### 5.2 Strength of evidence in MDD

In MDD, experimental and clinical findings converge around a stress–inflammation–BBB pathway, although direct mitochondrial evidence in brain endothelium remains limited. In chronic social-defeat stress, susceptible mice showed region-specific endothelial claudin-5 loss and abnormal nucleus-accumbens vasculature; claudin-5 downregulation permitted interleukin 6 entry, whereas antidepressant treatment promoted restoration and resilience (Menard et al., 2017). Endothelial TNF-α/NF-κB signaling and histone deacetylase 1 also mediated stress susceptibility, and inhibition restored claudin-5 (Dudek et al., 2020). Both studies included concordant human postmortem observations. A chronic mild-stress model similarly linked hippocampal leakage with epigenetic claudin-5 repression and peripheral TNF-α entry; claudin-5 restoration or EZH2 knockdown reduced barrier abnormalities and depression-like behavior (Sun et al., 2024).

These experimental findings provide a mechanistic context for the inflammatory and mitochondrial abnormalities reported in patients with MDD. Human studies have associated recent stress-induced depressive symptoms with an interaction between claudin-5 and IL-6 variants (Gal et al., 2023), and postmortem analyses have identified reduced hippocampal claudin-5 in major depression (Greene et al., 2020). At the cellular level, unmedicated patients with MDD showed reduced respiratory and glycolytic capacity in T cells (Gamradt et al., 2021), while a larger PBMC study reported altered expression of endoplasmic-reticulum stress, inflammasome, and mitochondrial-biogenesis genes, including context-dependent changes in NLRP3 and MFN2 (Munshi et al., 2025). These peripheral measurements do not establish mitochondrial dysfunction in BECs; however, they support a systemic immune-metabolic phenotype capable of increasing endothelial stress in susceptible patients.

Together, these findings support a testable model for an inflammatory MDD subgroup in which chronic stress and glucocorticoid dysregulation increase inflammatory and metabolic load, endothelial NF-κB and redox signaling, and region-specific junctional vulnerability. Corticosterone-induced hepatic complement C3, for example, suppressed endothelial claudin-5, increased nucleus-accumbens permeability, and promoted stress susceptibility in mice (Deng et al., 2026). Because mitochondrial function and BBB permeability have rarely been measured together in patients, this sequence remains a mechanistic framework rather than established causality in MDD.

### 5.3 Strength of evidence in BD

Human evidence in BD indicates abnormalities across inflammatory, oxidative, metabolic, and neurovascular domains, although their relationships vary according to mood state, illness stage, treatment exposure, and cardiometabolic status. Meta-analytic and clinical studies have reported increased oxidative damage and altered inflammatory mediators, with some immune abnormalities being more evident during mania or bipolar depression than during euthymia (Yuan et al., 2019; Skokou et al., 2023). These findings suggest that inflammatory and redox biology in BD may be dynamic rather than stable across all phases of illness.

Magnetic resonance spectroscopy and CSF studies provide *in vivo* evidence of altered cerebral bioenergetics. Higher lactate and relationships between lactate and glutathione have been reported in the dorsal anterior cingulate cortex of euthymic individuals with bipolar I disorder, supporting an association between cerebral energy metabolism and redox compensation (Soeiro-De-Souza et al., 2016). Broader mitochondrial evidence includes alterations in respiratory-chain activity, high-energy phosphate metabolism, OXPHOS, and cellular bioenergetics. However, the direction and magnitude of these changes vary according to the tissue examined, illness phase, medication exposure, and metabolic comorbidity (Skokou et al., 2023). These findingsdemonstrate altered bioenergetic measures in BD but do not establish that mitochondrial dysfunction causes mood episodes or BBB impairment.

Large-scale cerebrospinal-fluid proteomics provides human neurovascular evidence in BD. Across two cohorts, Göteson et al. (2026) quantified more than 2,000 proteins in 374 participants and identified 41 replicated case–control associations involving synaptic function, axon guidance, cell adhesion, complement signaling, and BBB integrity, particularly in BD type I. Longitudinal protein modules were also associated with severity, future mania, and symptom improvement. These signatures support altered immune and neurovascular biology but do not independently demonstrate increased permeability or mediation of clinical progression.

Other human studies have reported abnormalities in the QAlb, S100B, soluble adhesion molecules, MMPs, zonulin, and circulating tight-junction-related proteins in BD (Meixensberger et al., 2020; Zhao et al., 2022; Sheikh et al., 2023). These findings suggest that barrier-associated and systemic vascular processes may be altered in some patients, particularly those with recurrent episodes, inflammation, or metabolic comorbidity. However, circulating zonulin, claudin-5, S100B, and MMPs are indirect and incompletely specific indicators of BBB permeability. Their tissue origin cannot always be established, and changes may reflect intestinal permeability, systemic endothelial dysfunction, astrocytic stress, ECM remodeling, medication effects, or cardiometabolic disease.

Experimental evidence provides possible mechanisms through which oxidative and inflammatory stress could affect the NVU. Dopaminergic activity, mitochondrial ROS, inflammasome signaling, and endothelial adhesion molecules can modify vascular and mitochondrial functions under controlled conditions. Nevertheless, the observation of increased oxidative stress during mania does not demonstrate that dopamine-induced ROS disrupts the BBB in patients. Similarly, although lithium and valproate influence antioxidant defenses, mitochondrial signaling, and cellular resilience, their clinical efficacy has not been shown to depend on restoration of BBB integrity (Ortega et al., 2023; Skokou et al., 2023).

Mitochondrial, inflammatory, metabolic, and neurovascular abnormalities may coexist and fluctuate across BD states. Longitudinal studies combining DCE-MRI, paired cerebrospinal-fluid/serum measures, metabolic imaging, plasma mitochondrial biomarkers, and repeated assessment across mania, depression, and euthymia are needed before causal or state-dependent relationships can be established.

### 5.4 Strength of evidence in ASD

Human evidence suggests that mitochondrial and barrier-related abnormalities occur in subsets of individuals with ASD, but direct studies measuring both processes in the same participants remain scarce. Postmortem brain investigations currently provide the most relevant tissue-level evidence of altered BBB-associated biology. Fiorentino et al. (2016) reported changes in the expression of claudin-3, claudin-5, claudin-12, tricellulin, MMP-9, and other barrier- or neuroinflammation-related molecules in cerebral cortex and cerebellum from individuals with ASD. However, several tight-junction proteins were increased rather than reduced, and altered protein abundance does not necessarily indicate increased functional permeability. Postmortem studies are additionally limited by small sample sizes, clinical heterogeneity, medication exposure, cause of death, tissue quality, and inability to establish temporal order.

Separate postmortem and peripheral investigations provide evidence of mitochondrial abnormalities. Changes in respiratory-chain complex abundance or activity, oxidative damage, antioxidant defenses, and mitochondrial-related proteins have been reported in brain tissue from some individuals with ASD. Clinical studies using blood, muscle biopsies, fibroblasts, and lymphoblastoid cell lines have also identified altered lactate/pyruvate ratios, acylcarnitine profiles, mitochondrial respiration, membrane potential, and susceptibility to oxidative stress (Rossignol and Frye, 2011; Rose et al., 2014, 2018). A recent synthesis highlights alterations involving OXPHOS, calcium regulation, redox signaling, mitochondrial dynamics, mitophagy, and programmed cell death in ASD, while also emphasizing that it remains uncertain whether these abnormalities represent causes, consequences, or adaptive responses (Khaliulin et al., 2024).

Patient-derived cells support biologically distinct mitochondrial ASD subgroups. Lymphoblastoid cells, fibroblasts, neurons, and cerebral organoids show altered respiration, morphology, energy metabolism, calcium signaling, or stress responses; some exhibit compensatory hyper-respiration rather than uniform impairment (Rose et al., 2014; Frye et al., 2021). These models identify cell-autonomous phenotypes but cannot reproduce systemic metabolism, blood flow, immune trafficking, or the complete NVU.

The available evidence for barrier dysfunction in living individuals with ASD is substantially more limited. Circulating tight-junction-related proteins, intestinal permeability measures, inflammatory biomarkers, S100B, MMPs, and markers of endothelial activation have been investigated, but none independently provide a validated measure of cerebral BBB permeability. Intestinal barrier dysfunction should not be interpreted as evidence of BBB disruption, even when both processes coexist. At present, there is insufficient DCE-MRI or paired CSF/serum evidence to determine whether BBB permeability is consistently increased in clinically defined ASD subgroups.

Prenatal and perinatal immune-activation models provide an additional developmental hypothesis. Experimental maternal immune activation and early-life environmental exposures can modify fetal or offspring BBB properties, mitochondrial function, microglial maturation, and neurodevelopmental trajectories. These models suggest a potential route through which immune–metabolic stress could influence neurovascular development. However, evidence that this sequence occurs in humans or contributes directly to core ASD features remains limited.

ASD may include mitochondrial, oxidative, immune, metabolic, or barrier-associated subgroups, but these abnormalities have largely been measured separately. Current evidence does not establish that mitochondrial dysfunction causes BBB impairment or core ASD characteristics. Longitudinal human studies should integrate mitochondrial, endothelial, imaging, metabolic, genetic, gastrointestinal, and developmental measures within the same participants.

### 5.5 Strength of evidence in PTSD and stress-related disorders

Post-traumatic stress disorder has less direct BBB evidence than MDD. Experimental stress models show region- and susceptibility-dependent endothelial inflammatory signaling and junctional instability (Menard et al., 2017; Dudek et al., 2020; Samuels et al., 2023). Although not equivalent to PTSD, these models suggest how sympathetic activation, glucocorticoid dysregulation, and peripheral inflammation might affect the NVU after trauma. Mitochondrial ROS, quality-control failure, mtDNA signaling, and claudin-5 remodeling should therefore be viewed as candidate mediators, not mechanisms already demonstrated in PTSD.

Human mitochondrial findings in PTSD indicate stress-related cellular dysregulation but not BBB failure. Combat veterans with PTSD showed lower granulocyte mtDNA copy number than trauma-exposed controls (Bersani et al., 2016). In a larger cohort, circulating cell-free mtDNA did not differ before adjustment; lower levels emerged after accounting for diabetes, antidepressants, and age and were associated with greater glucocorticoid sensitivity (Blalock et al., 2024). Thus, circulating mtDNA is not uniformly elevated and is strongly influenced by metabolic comorbidity, medication, cellular composition, and hypothalamic–pituitary–adrenal-axis biology.

Cross-sectional human evidence provides a closer BBB link. Among 1,311 trauma-exposed participants and postmortem tissue from 100 decedents, trauma or PTSD-related measures were associated with selected claudin-5 methylation probes in blood and ventromedial prefrontal cortex; one probe related to plasma neurofilament light, and brain claudin-5 expression correlated with endothelial-cell proportion (Wolf et al., 2026). These findings support neurovascular involvement but do not demonstrate reduced claudin-5 protein or increased permeability. Trauma-related neuroendocrine and inflammatory signals may create parallel mitochondrial and endothelial vulnerabilities, which require simultaneous functional testing to establish convergence.

### 5.6 Strength of evidence in GAD

Human evidence in GAD supports a peripheral oxidative-stress phenotype but not a demonstrated mitochondrial–BBB pathway. Newly diagnosed, untreated patients showed higher serum 8-hydroxy-2′-deoxyguanosine and S100B, although S100B is not specific to BBB permeability (Oktay et al., 2024). In a 2025 treatment study, patients with GAD had higher malondialdehyde, lipid hydroperoxides, and cortisol and lower antioxidant-enzyme activity than controls; several markers changed with selective serotonin-reuptake-inhibitor treatment, and baseline malondialdehyde predicted response (Cui et al., 2025). These results link GAD to systemic redox dysregulation but do not localize mitochondrial injury to the NVU or establish BBB leakage. Direct DCE-MRI, cerebrospinal-fluid, and mitochondrial-functional studies are required.

### 5.7 Transdiagnostic clinical dimensions

A dimensional approach is appropriate because inflammatory, mitochondrial, metabolic, and neurovascular abnormalities do not map consistently onto diagnoses. Meta-analytic evidence shows overlapping inflammation across SZ, MDD, and BD, with magnitude influenced by clinical state, severity, medication, and metabolic comorbidity (Yuan et al., 2019; Thylur and Goldsmith, 2022). The proposed axis may therefore be more informative when related to cognition, fatigue, anhedonia, negative symptoms, cardiometabolic burden, biological aging, and treatment response than when treated as universal within a diagnosis.

#### 5.7.1 Cognitive impairment and fatigue

Cognition and fatigue are plausible convergence points for mitochondrial, inflammatory, and vascular biology. Across psychiatric populations, cognition has been associated with inflammatory proteins, adhesion molecules, perfusion, lactate, glutathione, and other bioenergetic measures (Sheikh et al., 2023; Solomon et al., 2025). Few studies, however, assess mitochondrial function, BBB permeability, neurovascular coupling, and cognition together. Mitochondrial–vascular coupling therefore remains a candidate explanation rather than an established mechanism. Medication, sleep, smoking, inactivity, and cardiometabolic disease should be modeled as confounders.

#### 5.7.2 Anhedonia and negative symptoms

Anhedonia represents one of the most extensively examined transdiagnostic dimensions associated with inflammatory and brain-network alterations. In 108 participants with recent-onset psychosis or depression, Lalousis et al. (2024) identified a potential anhedonic subgroup characterized by higher IL-6, S100B, and IL-1 receptor antagonist, lower interferon-γ, and altered functional connectivity involving the precuneus and posterior cingulate cortex. Nevertheless, the inflammatory and functional-connectivity models showed modest discriminatory performance, and circulating S100B is not specific to BBB permeability because it may reflect astroglial stress, altered barrier transport, or peripheral sources (Kozlowski et al., 2023). These findings support an association among anhedonia, inflammation, astroglial or barrier-related signaling, and functional connectivity but do not demonstrate that BBB dysfunction causes anhedonia.

Negative symptoms and motivational impairment have similarly been associated with inflammatory, metabolic, and rewards-circuit abnormalities across diagnostic groups. Experimental evidence suggests that inflammatory mediators can modify mitochondrial metabolism, dopamine-related signaling, and neurovascular function in rewards-associated regions (Kim et al., 2019). However, direct human evidence demonstrating that mitochondrial dysfunction within the BBB mediates negative symptoms remains unavailable. Anhedonia and negative symptoms should therefore be regarded as candidate dimensions for multimodal mitochondrial–neurovascular studies rather than direct consequences of barrier disruption.

#### 5.7.3 Illness severity and treatment response

Recent human evidence supports illness severity as a biologically informative transdiagnostic dimension. Solomon et al. (2025) analyzed longitudinal symptom trajectories and cognitive performance in 443 participants with SZ, BD, or MDD and identified two clusters that differed in illness severity but not in diagnostic composition. Nineteen serum proteins differed between the clusters, with enrichment of immune, inflammatory, neurodevelopmental, and brain-plasticity pathways. These findings suggest that systemic inflammatory profiles may be more closely associated with overall illness burden than with a specific diagnosis. However, serum proteins cannot establish CNS inflammation, BBB permeability, or mitochondrial dysfunction, and the study does not demonstrate that inflammation causes greater disease severity.

Persistent inflammation, oxidative stress, metabolic disease, and mitochondrial abnormalities have each been associated with poorer treatment outcomes in psychiatric populations (Miller, 2020; Thylur and Goldsmith, 2022). Nevertheless, it remains uncertain whether BBB dysfunction, altered cerebral drug transport, or impaired mitochondrial adaptation mediates these associations. Treatment resistance should therefore be considered a candidate phenotype for biomarker-enriched studies combining inflammatory markers, plasma or PBMC mitochondrial measurements, metabolic assessment, and neurovascular imaging.

#### 5.7.4 Accelerated biological aging and metabolic comorbidity

Psychiatric disorders frequently coexist with obesity, insulin resistance, dyslipidemia, hypertension, smoking, physical inactivity, and sleep or circadian disruption. These factors can independently modify inflammatory signaling, mitochondrial function, endothelial physiology, cerebral perfusion, and cognitive performance. Systemic adhesion molecules have been associated with severe mental illness, but their vascular origin cannot be localized specifically to the BBB (Sheikh et al., 2023). Similarly, altered circulating acylcarnitines and related metabolic markers have been associated with depressive symptoms, but these measurements reflect systemic metabolism and cannot be assumed to represent mitochondrial activity within the brain or NVU (Zacharias et al., 2021).

The mitochondrial–BBB axis integrates overlapping transdiagnostic vulnerabilities rather than constituting a single pathway to psychiatric symptoms. Human studies support associations among inflammatory activity, mitochondrial and vascular measures, cognition, anhedonia, fatigue, and illness severity but not a causal sequence. Longitudinal multimodal studies should combine neurovascular imaging, magnetic resonance spectroscopy, peripheral mitochondrial assays, inflammation, metabolism, and dimensional clinical assessment.

Table 1 summarizes disorder-specific and transdiagnostic evidence linking mitochondrial alterations, BBB or neurovascular abnormalities, and inflammatory features. Key references are mentioned in Supplementary Table 1.

**TABLE 1:** Clinical and preclinical evidence relevant to the mitochondrial–BBB axis across psychiatric disorders.

Disorder	Clinical evidence (human)	Preclinical evidence	Cautious synthesis	Critical gap
SZ	Postmortem changes in CLDN5/CLDN12/ZO-1 and endothelial–astrocytic ultrastructure; DCE-MRI evidence of higher regional leakage; QAlb, S100B, adhesion-molecule, redox, lactate, glutathione, and circulating mtDNA abnormalities in subsets; patient-derived endothelial cells/organoids show barrier-related phenotypes.	iPSC-derived BECs and organoids model endothelial and mitochondrial vulnerability; 22q11.2 deletion iBMEC and mouse studies show improvement of mitochondrial/barrier readouts with bezafibrate; inflammatory and oxidative endothelial models provide pathway-level support.	Human data support coexisting neurovascular, inflammatory, redox, and mitochondrial abnormalities in subgroups. Experimental systems make a mitochondrial–endothelial link plausible but do not establish that it initiates SZ or symptoms.	Few longitudinal studies measure BBB permeability and mitochondrial function in the same patients; major medication, smoking, and metabolic confounding.
MDD	Human postmortem studies support region-specific CLDN5 abnormalities; stress-related CLDN5/IL-6 genetic interaction; altered T-cell/PBMC respiration and immune–mitochondrial gene expression; circulating inflammatory, oxidative, and mtDNA-related markers in subsets. Direct *in vivo* BBB–mitochondrial co-measurement remains rare.	Chronic social-defeat and unpredictable-stress models show region- and susceptibility-dependent CLDN5 loss, endothelial TNF-α/NF-κB or complement signaling, and permeability changes; manipulation of CLDN5-related pathways modifies stress phenotypes.	MDD has relatively strong experimental support for a stress–inflammation–endothelial sequence and supportive human tissue evidence. The mitochondrial component in human brain endothelium remains mostly indirect.	Need multimodal longitudinal studies across episodes/remission, with DCE-MRI or QAlb plus mitochondrial assays and metabolic covariates.
BD	CSF proteomics identifies replicated complement, cell-adhesion, synaptic, and BBB-related signatures; MRS and CSF studies show lactate/redox abnormalities; QAlb, S100B, soluble adhesion molecules, MMPs, and circulating junctional proteins vary in subsets and by clinical state.	Oxidative, dopaminergic, inflammatory, and mitochondrial models show that ROS/inflammasome signaling can alter endothelial and NVU function; mood stabilizers modulate mitochondrial and antioxidant pathways in experimental systems.	Inflammatory, bioenergetic, and neurovascular signals may coexist and fluctuate with mood state, illness stage, and metabolic burden; their temporal and causal relationships are unresolved.	Repeated within-person measurements across mania, depression, and euthymia are scarce; circulating barrier markers lack brain specificity.
ASD	Postmortem cortex/cerebellum show altered barrier-related proteins and transcripts, with mixed directions; brain, blood, muscle, fibroblast, and lymphoblast studies identify respiratory, redox, membrane-potential, lactate/pyruvate, and acylcarnitine abnormalities in subsets. Direct DCE-MRI/QAlb evidence is insufficient.	Patient-derived fibroblast, neuronal, lymphoblast, and organoid models reveal heterogeneous mitochondrial phenotypes; maternal immune-activation and developmental models link immune–metabolic stress to BBB and NVU development.	Mitochondrial and barrier-associated abnormalities are supported in different samples and subgroups, but they have seldom been demonstrated together and cannot be inferred from intestinal permeability.	Need developmentally stratified human studies combining direct BBB measures, mitochondrial phenotyping, genetics, and gastrointestinal assessments.
PTSD and stress-related disorders	Combat-exposed cohorts report lower granulocyte mtDNA copy number and context-dependent plasma cf-mtDNA associations with symptoms/glucocorticoid sensitivity; emerging large-cohort and postmortem data support stress-related endothelial/CLDN5 biology. Direct human BBB evidence remains limited.	Chronic social/environmental stress models show susceptibility- and region-dependent vascular-cell reprogramming, inflammatory signaling, and junctional instability; mitochondrial redox and quality-control pathways are plausible mediators.	Human mitochondrial findings support stress-related cellular dysregulation but are not specific evidence of BBB failure. Experimental stress biology should not be treated as a direct PTSD model.	Prospective post-trauma cohorts should integrate hormones, sleep, inflammation, mitochondrial assays, DCE-MRI/QAlb, and symptom trajectories.
GAD	Serum studies report oxidative DNA/lipid damage, altered antioxidant enzymes, cortisol, and nonspecific S100B changes (Oktay et al., 2024; Cui et al., 2025).	Stress/anxiety models implicate mitochondrial ROS, hypothalamic–pituitary–adrenal signaling, endothelial inflammation, and junctional vulnerability, but are not GAD-specific.	Human evidence supports peripheral redox dysregulation, not direct mitochondrial dysfunction within the NVU or BBB leakage.	DCE-MRI, paired cerebrospinal-fluid/serum measures, and mitochondrial assays are needed in well-characterized GAD cohorts.
Transdiagnostic dimensions	Inflammation, anhedonia, cognitive impairment, fatigue, insulin resistance, and vascular risk cut across diagnoses; multimodal studies associate cytokines, endothelial markers, S100B, metabolic measures, and imaging phenotypes with symptom dimensions.	Across models, mtROS, impaired mitophagy, mtDNA danger signaling, NF-κB/NLRP3 activation, calcium dysregulation, MMP activity, and junctional remodeling form recurrent experimental pathways.	A mitochondrial–BBB endotype may be more informative than diagnosis alone, but current evidence supports stratification hypotheses rather than a validated clinical subtype.	Prospective replication, standardized assays, prespecified multimodal panels, and external validation of endotypes are required.

Clinical evidence is prioritized and separated from experimental evidence. The convergence column is deliberately phrased as an interpretation rather than a causal conclusion. Representative studies are described in sections “5.1 Strength of evidence in SZ”–“5.6 Strength of evidence in GAD” and Supplementary Table 1. ASD, autism spectrum disorder; BD, bipolar disorder; BEC, brain endothelial cell; BBB, blood-brain barrier; cf-mtDNA, cell-free mitochondrial DNA; CLDN, claudin; CSF, cerebrospinal fluid; DCE-MRI, dynamic contrast-enhanced magnetic resonance imaging; GAD, generalized anxiety disorder; iBMEC, induced brain microvascular endothelial cell; iPSC, induced pluripotent stem cell; MDD, major depressive disorder; MMP, matrix metalloproteinase; mtDNA, mitochondrial DNA; NVU, neurovascular unit; PBMC, peripheral blood mononuclear cell; PTSD, post-traumatic stress disorder; QAlb, CSF/serum albumin quotient; S100B, S100 calcium-binding protein B; SZ, schizophrenia; TNF-α, tumor necrosis factor alpha; ZO-1, zonula occludens-1.

## 6 Biomarkers of mitochondrial and BBB dysfunction

### 6.1 Peripheral inflammatory markers

Cytokines, chemokines, C-reactive protein (CRP), and soluble adhesion molecules reflect systemic inflammation but are influenced by adiposity, infection, medication, smoking, metabolic syndrome, and vascular disease; their main value is therefore stratification (Poletti et al., 2024). TNF-α, IL-6, and IL-1β may accompany endothelial activation and BBB vulnerability, but peripheral concentrations do not prove barrier disruption. Interpretation should combine them with more direct measures such as the QAlb, permeability imaging, or BBB-associated proteins (Cui et al., 2024; Ye X. et al., 2025).

In MDD, CRP is one of the most clinically useful inflammatory markers because it is inexpensive, reproducible and has already been used to identify treatment-relevant subgroups (Raison et al., 2013). In SZ, peripheral inflammation also appears clinically meaningful, although highly heterogeneous across patients and illness stages (Chumakov et al., 2022). Several studies have associated CRP and IL-6 with negative symptoms, cognitive impairment, alterations in white matter integrity and functional outcomes in patients with SZ and BD, but these markers remain non-specific and may be influenced by smoking, adiposity, infection, medication exposure, and cardiometabolic comorbidity (Boozalis et al., 2018; Sæther et al., 2022; Skokou et al., 2023).

On the other hand, chemokines, such as C-C motif chemokine ligand 2 (CCL2), and soluble adhesion molecules, such as soluble ICAM-1 (sICAM-1) and soluble VCAM-1 (sVCAM-1), provide information about vascular endothelial activation. Recent studies suggest that elevated sVCAM-1 levels in the blood precede changes in BBB permeability detectable by imaging, suggesting that these markers could act as early signals of vascular vulnerability in states of chronic stress (Dion-Albert et al., 2022). In severe mental illness, increased sICAM-1 has been reported (Sheikh et al., 2023), and CSF studies in SZ also suggest altered adhesion molecule biology (Meixensberger et al., 2021); nevertheless, these markers are not BBB-specific because they can originate from systemic vascular endothelium as well as CNS-associated compartments.

### 6.2 BBB-related biomarkers

The QAlb remains the clinical reference measure of barrier permeability and is elevated in some SZ and BD subgroups, but lumbar puncture limits routine use (Konstantinou and Warsh, 2026). S100B, MMP-9, ICAM-1, VCAM-1, and extracellular vesicles are less invasive but reflect overlapping astrocytic, endothelial, matrix-remodeling, and systemic vascular processes rather than permeability alone (Futtrup et al., 2020; Meixensberger et al., 2020). Sex-related QAlb differences further emphasize the need for appropriate reference ranges (Meixensberger et al., 2020).

In recent years, elevated circulating S100B has been reported across major psychiatric disorders; however, S100B can originate from astrocytes, adipose tissue and other peripheral sources, making it more appropriate as a broad glial–vascular stress marker than as an autonomous BBB permeability measure (Kozlowski et al., 2023). Therefore, more recent studies suggest that S100B, in combination with markers of axonal damage such as neurofilament light chain (NfL), can predict the trajectory of neuroprogression in severe affective disorders (Comai, 2025).

At the structural level, serum detection of claudin-5 has been investigated as a candidate biomarker of physical disruption of the BBB. Research in chronic stress models and human tissues demonstrates that claudin-5 degradation, often mediated by MMP-9 activation, is a hallmark of major depression (Greene et al., 2020; Sun B. et al., 2025). MMP-9 is relevant because it links inflammation, ECM remodeling and tight junction disruption. In psychiatric biomarker studies, MMPs are best interpreted as indicators of vascular remodeling or inflammatory proteolysis rather than specific markers of BBB leakage; their interpretation is strengthened when combined with other endothelial or barrier markers (Futtrup et al., 2020).

Endothelial activation can be monitored using adhesion molecules such as ICAM-1 and VCAM-1. A key study identified that increased VCAM-1 in the cerebral vasculature of females under social stress is sufficient to induce permeability and depressive-like behaviors, positioning these molecules not only as markers but also as drivers of the clinical phenotype (Dion-Albert et al., 2022).

Finally, the field of extracellular vesicles (EVs) is emerging as a type of liquid biopsy for the brain. EVs derived from endothelial cells and astrocytes allow for the analysis of the protein, microRNA, and metabolite content that these cells release into the general circulation (Ye X. et al., 2025). Protein analysis in astrocytic EVs has revealed altered levels of complement and inflammasome markers in patients with psychosis, offering a window to monitor CNS inflammation without the need for invasive procedures (Comai, 2025; Ye X. et al., 2025).

### 6.3 Mitochondrial biomarkers

Circulating mtDNA and mitochondrial DNA copy number (mtDNAcn): Recent studies have shown that circulating cf-mtDNA may serve simultaneously as a marker of mitochondrial injury and a mediator of neuroinflammatory processes relevant to BBB dysfunction (Blalock et al., 2024). In SZ, plasma cf-mtDNA has been studied in relation to cognitive deficit, while first-episode SZ studies suggest that cf-mtDNA may change with clinical progression or antipsychotic treatment (Kumar et al., 2018; Ouyang et al., 2021); In whole-blood mtDNA copy number studies in psychosis further show that mtDNA measures can be influenced by cellular composition and comorbidity, so platelet/leukocyte correction or cell-specific assays are important (Kumar et al., 2018).

Lactate and pyruvate: An elevated lactate/pyruvate ratio suggests a metabolic shift toward anaerobic glycolysis due to an inefficient electron-transport chain (ETC), a recurring finding in patients with BD during manic episodes (Giménez-Palomo et al., 2024; Berk et al., 2025). In patients with BD and SZ, elevated CSF lactate was reported, supporting altered central energy metabolism. However, lactate is highly sensitive to peripheral metabolism, exercise, stress, sampling conditions and medication effects; therefore, it is most useful when interpreted alongside pyruvate, glucose, TCA-cycle intermediates or magnetic resonance spectroscopy-based energy measures (Regenold et al., 2008; Cao et al., 2025).

Acylcarnitines reflect fatty-acid transport and β-oxidation. Distinct profiles have been reported in ASD with developmental regression (Frye et al., 2024) and associated with depressive symptoms in a 1,411-participant metabolomic study (Zacharias et al., 2021). During major depressive episodes, medium- and long-chain acylcarnitines were reduced and partly normalized after six months of treatment, with several metabolites inversely related to symptom severity (Ait Tayeb et al., 2024). Targeted metabolomics before and after escitalopram also showed shifts in short-, medium-, and long-chain acylcarnitines, consistent with altered β-oxidation during treatment (MahmoudianDehkordi et al., 2021). These patterns are informative but remain sensitive to diet, medication, sex, and systemic metabolism.

Respiratory chain activity and cellular bioenergetics: In major depression, PBMCs mitochondrial respiration was reduced and correlated with depressive subsymptoms and severity (Karabatsiakis et al., 2014), supporting PBMCs bioenergetics as a clinically informative readout. Additionally, NAD^+^-related metabolites are promising because NAD^+^ regulates mitochondrial respiration, redox balance, poly(ADP-ribose) polymerase (PARP) activity, DNA repair and sirtuin-dependent stress responses. Recent metabolomic studies in treatment-refractory depression and stress-related models highlight alterations in energy and NAD^+^-linked pathways, suggesting that NAD^+^ metabolism may help capture mitochondrial resilience or exhaustion (Pan et al., 2023; Chen et al., 2024). Importantly, a decreased NAD+/NADH ratio has been proposed as a signature of metabolic vulnerability in SZ (Cuklanz et al., 2026).

Reactive oxygen species -related markers and glutathione. Brain glutathione can be measured by MRI, and studies in psychosis (Murray et al., 2024) and depression (Bell et al., 2025) suggest that glutathione alterations may relate to clinical stage, symptoms and methodological differences. However, ROS-related markers are influenced by inflammation, diet, smoking, metabolic disease and medications; therefore, they should be interpreted as redox-state indicators rather than mitochondrial-specific markers.

### 6.4 Neuroimaging and functional readouts

Unlike blood-based markers, imaging can localize BBB permeability, neuroinflammatory activity, energy-metabolic abnormalities, perfusion and neurovascular coupling within specific brain regions and circuits.

BBB permeability imaging: In SZ-spectrum disorders, Moussiopoulou et al. (2025) reported higher BBB leakage using DCE-MRI compared with healthy controls, supporting the feasibility of quantifying barrier dysfunction in living psychiatric patients. This is highly relevant for biomarker panels because DCE-MRI may provide a direct neurovascular phenotype that can be integrated with inflammatory, mitochondrial and cognitive measures (Moussiopoulou et al., 2025).

Positron emission tomography (PET) markers of neuroinflammation: PET imaging can provide molecular information about neuroinflammatory processes, particularly through translocator protein (TSPO) ligands that are often interpreted as markers of activated microglia and astrocytes. However, TSPO PET findings in psychiatric disorders are heterogeneous; for example, several SZ studies have reported unchanged or reduced TSPO binding rather than consistent elevation (Conen et al., 2020). A recent study comparing TSPO binding in depression with and without suicide attempt history found that elevated TSPO total distribution volume was associated with more pronounced daily suicidal ideation and negative affect in the context of real-world stressors (Herzog et al., 2025), illustrating the value of moving from broad diagnostic contrasts toward clinically meaningful inflammatory phenotypes.

Magnetic resonance spectroscopy (MRS) non-invasively assesses metabolites related to bioenergetics and redox balance. Studies in SZ report elevated lactate and illness-duration-related regional differences in lactate and neurotransmitter metabolites (Rowland et al., 2016; Wijtenburg et al., 2021). In euthymic BD, dorsal anterior cingulate lactate correlated with glutathione, suggesting coupled energy and redox adaptation (Soeiro-De-Souza et al., 2016). A meta-analysis in depression found lower occipital glutathione (g = −0.98; 95% CI, −1.45 to −0.50; *P* < 0.001), although individual-level utility remains unresolved (Bell et al., 2025). These readouts can complement BBB imaging but do not localize abnormalities specifically to mitochondria or the NVU.

Perfusion and neurovascular coupling measures: Perfusion imaging can capture vascular function downstream of BBB and mitochondrial stress. Arterial spin labeling (ASL)-MRI has shown altered cerebral perfusion in major depression, and Lui et al. (2009) reported focal perfusion abnormalities in depressive disorders. Although ASL does not measure BBB permeability directly, it provides a functional vascular readout that may complement DCE-MRI and metabolic imaging. Importantly, neurovascular coupling approaches that integrate resting-state functional MRI and ASL are particularly relevant because they connect neural activity with cerebral blood flow regulation. In fact, recent work in adolescent MDD reported altered neurovascular coupling using combined resting-state functional MRI and ASL, suggesting that vascular–neural coordination may be altered early in mood disorders (Yin et al., 2026).

### 6.5 Multimodal biomarker panels

A multimodal panel combining inflammatory, BBB-related, mitochondrial, metabolic, and imaging measures may support biological stratification. Candidate measures and their limitations are summarized in Table 2 and Supplementary Table 2.

**TABLE 2:** Prioritized multimodal biomarkers for studying the mitochondrial-BBB axis.

Domain/ priority	Preferred biomarker or readout	Specimen/ modality	Best-supported use	Main limitation
Barrier permeability — core	CSF/serum albumin quotient (QAlb); DCE-MRI permeability (e.g., Ktrans)	Paired CSF + serum; MRI	QAlb provides a clinically established paired-fluid index; DCE-MRI provides regional *in vivo* permeability mapping. Use together when feasible.	QAlb is invasive and also reflects the blood–CSF barrier; DCE-MRI protocols, thresholds, and contrast exposure require harmonization.
Endothelial activation — core	sICAM-1, sVCAM-1, CCL2/MCP-1; endothelial-derived EV cargo	Plasma/serum; extracellular vesicles	Captures leukocyte recruitment and endothelial activation; EV cargo may increase cell-type resolution.	Not brain-specific; EV isolation, cellular attribution, and normalization remain heterogeneous.
Inflammation — core	hsCRP, IL-6, TNF-α; optional IL-1β/complement panel	Serum/plasma; CSF when available	Identifies inflammatory endotypes and contextualizes endothelial and mitochondrial readouts.	Strong effects of adiposity, infection, smoking, medication, circadian state, and assay platform.
Junctional/matrix remodeling — complementary	CLDN5, OCLN, ZO-1; MMP-2/MMP-9	Plasma/serum, CSF, EVs; tissue	Assesses junctional-protein abundance or proteolytic remodeling; strongest when paired with direct permeability measures.	Circulating concentrations do not establish cerebral origin or functional BBB leakage.
Astroglial/axonal injury — complementary	S100B ± NfL	Serum/plasma; CSF	Provides injury context and may improve interpretation of neurovascular abnormalities.	Neither marker is BBB-specific; S100B has extracerebral sources and NfL primarily reflects axonal injury.
Mitochondrial DNA dynamics — core	Plasma/CSF cf-mtDNA; blood-cell or PBMC mtDNA copy number	Platelet-poor plasma, CSF, isolated cells	Indexes mitochondrial injury/danger signaling and cellular mitochondrial abundance or adaptation.	Highly sensitive to platelet contamination, cell composition, extraction, storage, exercise, medication, and metabolic disease.
Cellular bioenergetics — core	Basal/maximal oxygen-consumption rate, ATP-linked respiration, spare respiratory capacity, ΔΨm, mitochondrial ROS	Fresh PBMCs or defined immune-cell subsets	Directly characterizes functional mitochondrial phenotypes and target engagement.	Requires viable cells, rapid processing, cell-subset control, and interlaboratory standardization.
Metabolic/redox state — complementary	Lactate/pyruvate, acylcarnitines, NAD+/NADH, glutathione (GSH)	Plasma/serum, CSF; metabolomics; MRS for brain GSH/lactate	Captures bioenergetic shift, substrate use, redox balance, and central–peripheral concordance.	Affected by diet, fasting, exercise, hypoxia, collection handling, systemic metabolism, and spectral resolution.
Mitochondrial quality control — emerging	PINK1, Parkin, LC3-II/p62; DRP1, MFN1/2, OPA1; mitophagic flux where feasible	PBMCs, EVs, patient-derived cells	Tests mitophagy and mitochondrial-dynamics mechanisms and pharmacodynamic response.	Static protein abundance may not represent flux; limited psychiatric and BBB-specific clinical validation.
Integrated imaging — complementary	ASL perfusion, cerebrovascular reactivity, MRS lactate/GSH, TSPO-PET	MRI/MRS/PET	Adds perfusion, neurovascular coupling, cerebral metabolic/redox, and neuroinflammatory context to permeability measures.	These readouts are not direct measures of BBB integrity; TSPO genotype and cellular specificity affect interpretation.
Stress-response signals — emerging	FGF21 and GDF15	Serum/plasma	May identify systemic mitochondrial-stress phenotypes and support exploratory stratification.	Low CNS and BBB specificity; strong effects of age, exercise, liver/kidney disease, and metabolic health.

No single marker is diagnostic or sufficiently BBB-specific; the proposed use is as a harmonized research panel combining direct or near-direct barrier measures with mitochondrial, inflammatory, endothelial, metabolic, and clinical readouts. ASL, arterial spin labeling; BBB, blood-brain barrier; CCL2, C-C motif chemokine ligand 2; cf-mtDNA, cell-free mitochondrial DNA; CLDN5, claudin-5; CSF, cerebrospinal fluid; DCE-MRI, dynamic contrast-enhanced magnetic resonance imaging; DRP1, dynamin-related protein 1; EV, extracellular vesicle; FGF21, fibroblast growth factor 21; GDF15, growth differentiation factor 15; GSH, glutathione; hsCRP, high-sensitivity C-reactive protein; ICAM-1, intercellular adhesion molecule 1; IL, interleukin; LC3-II, microtubule-associated protein 1A/1B-light chain 3-II; MCP-1, monocyte chemoattractant protein 1; MFN1/2, mitofusin 1/2; MMP, matrix metalloproteinase; MRI, magnetic resonance imaging; MRS, magnetic resonance spectroscopy; mtDNAcn, mitochondrial DNA copy number; NAD+/NADH, oxidized/reduced nicotinamide adenine dinucleotide; NfL, neurofilament light chain; OCR, oxygen-consumption rate; OCLN, occludin; OPA1, optic atrophy 1; PBMC, peripheral blood mononuclear cell; PET, positron-emission tomography; PINK1, PTEN-induced kinase 1; QAlb, CSF/serum albumin quotient; ROS, reactive oxygen species; sICAM-1, soluble ICAM-1; sVCAM-1, soluble VCAM-1; TSPO, 18-kDa translocator protein; ZO-1, zonula occludens-1; ΔΨm, mitochondrial membrane potential.

## 7 Therapeutic and translational implications

### 7.1 Mechanism-based targeting of mitochondrial dysfunction

Therapeutic translation of the mitochondrial–BBB framework should move beyond the non-specific concept of “antioxidant treatment.” The relevant interventions act at distinct levels: mitochondrial ROS production, cardiolipin and respiratory-chain organization, the coenzyme Q pool, glutathione-dependent redox buffering, NAD+-dependent signaling, selective removal of damaged mitochondria, and PGC-1α-regulated mitochondrial biogenesis. These mechanisms may converge on endothelial ATP availability, redox-sensitive tight-junction regulation, inflammatory signaling, and neurovascular coupling. However, the evidentiary chain remains incomplete: most studies have measured symptoms or mitochondrial outcomes without directly quantifying BBB permeability, whereas many BBB-protective findings arise from non-psychiatric models. The agents below should therefore be regarded as mechanism-informed candidates for biomarker-enriched trials rather than established treatments for BBB dysfunction in psychiatric disorders.

#### 7.1.1 Mitochondria-targeted antioxidants: MitoQ and SS-31/elamipretide

MitoQ is a triphenylphosphonium-conjugated ubiquinone that accumulates within polarized mitochondria and is reduced to an antioxidant-active ubiquinol form. By lowering mitochondrial lipid peroxidation and ROS amplification, MitoQ could attenuate redox-sensitive NF-κB/NLRP3 signaling, preserve nitric-oxide bioavailability, and limit oxidative modification of junctional and cytoskeletal proteins in BECs. Preclinical BBB protection has been reported in acute neurological injury, and recent work shows that MitoQ can stabilize PINK1 and enhance mitophagy in cells carrying the m.3243A > G mitochondrial variant (Cao et al., 2026). Nevertheless, no adequately powered randomized trial has established antidepressant, antipsychotic, or PTSD efficacy, and benefit may depend on residual mitochondrial membrane potential because cationic uptake declines in severely depolarized mitochondria. Dose, tissue target engagement, and potential pro-oxidant effects at excessive concentrations must therefore be measured rather than assumed.

Szeto–Schiller peptide 31 (SS-31; elamipretide) is a cell-permeable aromatic–cationic tetrapeptide that associates with cardiolipin on the inner mitochondrial membrane. Its proposed effects include stabilization of cristae architecture, improved electron-transfer efficiency, reduced electron leak, and preservation of ATP production. This profile is particularly relevant to BBB endothelial cells, which have high energetic demands. In aged mice, elamipretide reduced cerebral microhemorrhage burden in a preclinical screen of cerebromicrovascular protection (Patai et al., 2025), and a 2026 post-cardiac-arrest study reported reduced microglial ferroptosis and inflammatory polarization (Jiang et al., 2026). These data support vascular and neuroimmune plausibility but cannot be interpreted as psychiatric efficacy. Trials in MDD, PTSD, BD, or SZ should first demonstrate CNS exposure and effects on mitochondrial respiration or BBB-related biomarkers.

#### 7.1.2 CoQ10 and redox support: melatonin and n-acetylcysteine

Coenzyme Q10 (CoQ10) differs from MitoQ because it is an endogenous electron carrier and lipid-phase antioxidant rather than a mitochondria-targeted synthetic conjugate. It may support complexes I/II–III electron transfer while limiting membrane lipid oxidation, but oral bioavailability, formulation, baseline CoQ10 status, and penetration into the CNS are variable. Two recent meta-analyses provide the strongest contemporary clinical signal among the interventions considered here. Akwan et al. (2025) found limited, scale- and subgroup-dependent improvement in depressive symptoms, especially with 100–200 mg/day for 6–8 weeks, but not a clear anxiolytic effect. (Magalhães et al., 2026) reported a moderate pooled reduction in depressive symptoms across five trials, although only two trials enrolled primary depressive disorders and the remaining studies involved depression associated with medical illness. Thus, CoQ10 is promising as adjunctive therapy, particularly in patients with metabolic or redox abnormalities, but the evidence is too heterogeneous to recommend it as a diagnosis-wide treatment.

Melatonin is both a chronobiotic acting through melatonin receptor 1/2 (MT1/MT2) and a receptor-independent redox regulator that can reach intracellular and mitochondrial compartments. It can reduce ROS, influence sirtuin 1 (SIRT1)/peroxisome proliferator-activated receptor gamma coactivator 1 alpha (PGC-1α) signaling and mitochondrial biogenesis and suppress MMP-9 and inflammatory pathways implicated in BBB permeability. Its psychiatric relevance is greatest when mitochondrial stress co-occurs with insomnia or circadian disruption; however, improvement in sleep may mediate symptom change independently of direct mitochondrial or BBB rescue. Recent synthesis emphasizes mitochondrial and neuroprotective actions (Zhang Z. et al., 2024), but disorder-specific evidence linking melatonin treatment to BBB restoration remains absent. Future trials should separate chronobiological from mitochondrial effects through actigraphy or dim-light melatonin onset, redox markers, and BBB-sensitive outcomes.

N-acetylcysteine (NAC) replenishes cysteine for glutathione synthesis, modulates cystine–glutamate exchange, and may reduce oxidative and inflammatory stress that secondarily impairs mitochondria and the BBB. Clinical findings are inconsistent across diagnoses. Importantly, a 2025 randomized trial in 182 participants with co-occurring PTSD and alcohol use disorder found that NAC 2,400 mg/day was well-tolerated but did not outperform placebo plus cognitive behavioral therapy for PTSD or alcohol outcomes (Back et al., 2025). This negative result argues against presenting NAC as a general treatment for PTSD. A more defensible precision strategy would test whether low glutathione, high oxidative stress, inflammation, or metabolic comorbidity identifies a responsive subgroup and whether changes in those markers mediate clinical improvement.

#### 7.1.3 NAD+ restoration: nicotinamide riboside and other NAD+ enhancers

Oxidized nicotinamide adenine dinucleotide (NAD+) availability connects redox metabolism with sirtuin activity, poly (ADP-ribose) polymerase (PARP)-dependent DNA repair, inflammatory signaling, mitophagy, and PGC-1α-mediated biogenesis. Nicotinamide riboside (NR) and nicotinamide mononucleotide (NMN) increase the systemic NAD+ pool, although recent mechanistic work indicates that their utilization involves enterohepatic conversion and inter-organ metabolism rather than simple direct uptake into every target tissue (Yaku et al., 2025). In a 2025 crossover trial in older adults with subjective cognitive decline or mild cognitive impairment, NR at 1 g/day for eight weeks was safe and reduced plasma p-tau217 but did not improve cognition (Wu et al., 2025). This provides human safety and biomarker evidence outside psychiatry, not proof of antidepressant or BBB efficacy.

#### 7.1.4 Mitophagy and mitochondrial-biogenesis strategies

Selective mitophagy prevents persistence of mitochondria that release ROS and oxidized mtDNA. Recent depression models strengthen the mechanistic link: chronic mild stress disrupted mitophagy and mitochondrial status in rat frontal cortex (Ulecia-Morón et al., 2025); NDUFB9 overexpression promoted mitophagy and reduced CUMS-induced depressive-like behavior (Sun Y. et al., 2025); and astrocytic HDAC7 impaired PINK1-dependent mitophagy and induced depression-like phenotypes (Yue et al., 2025). These findings identify PINK1/Parkin, AMPK–SIRT1, and related quality-control pathways as candidates, but direct systemic activation of mitophagy could be harmful if excessive or cell-non-specific.

PGC-1α activation offers a complementary strategy by increasing mitochondrial biogenesis, oxidative metabolism, and antioxidant capacity. Exercise remains the most clinically feasible pleiotropic activator; in a 2025 hypertensive mouse model it enhanced mitochondrial biogenesis and rescued structural BBB abnormalities (Chang et al., 2025). The strongest direct bridge to psychiatric vulnerability comes from 22q11.2 deletion syndrome: bezafibrate, a pan-peroxisome proliferator-activated receptor (PPAR) agonist that induces mitochondrial biogenesis, improved mitochondrial function and BBB integrity in patient-derived BECs and a mouse model, and corrected a social-memory deficit correlated with BBB function (Crockett et al., 2025). This study supports causal plausibility but not routine bezafibrate use in psychiatric patients. PPAR-related adverse effects, lipid phenotype, hepatic monitoring, and interaction with psychotropic-associated metabolic disease must be considered.

### 7.2 Personalized and stratified psychiatry

One of the principal challenges preventing the translation of mechanistic discoveries into psychiatric practice is the remarkable biological heterogeneity that exists within conventional diagnostic categories. Individuals fulfilling criteria for the same Diagnostic and Statistical Manual of Mental Disorders (DSM) diagnosis may differ substantially in the magnitude of systemic inflammation, mitochondrial bioenergetic capacity, oxidative stress, endothelial function, BBB integrity, and metabolic health. Consequently, the mitochondrial–BBB axis should not be regarded as a universal pathogenic mechanism operating equally across all patients but rather as a biological framework capable of identifying clinically meaningful subgroups with distinct pathophysiological drivers and therapeutic vulnerabilities. This perspective is consistent with the growing field of precision psychiatry, which seeks to complement symptom-based diagnosis with molecular, cellular and physiological signatures that better reflect disease biology rather than clinical presentation alone (Colombo et al., 2024; Fabbri, 2025; Miller, 2025).

#### 7.2.1 A multidimensional biological stratification framework

Inflammatory endotype: The first and currently best-supported layer is the inflammatory signature. Numerous studies indicate that only a subset of psychiatric patients exhibits persistent activation of innate immunity characterized by increased circulating CRP, IL-6, TNF-α, IL-1β, chemokines and other inflammatory mediators. These patients frequently present greater symptom severity, cognitive dysfunction, fatigue, metabolic abnormalities and poorer response to conventional monoaminergic treatments. Therefore, inflammatory biomarkers may serve primarily as stratification tools rather than diagnostic markers, helping identify individuals in whom immune–metabolic mechanisms contribute substantially to disease biology (Goldsmith et al., 2016; Köhler-Forsberg et al., 2017; Miller, 2025; Paganin, 2025).

Neurovascular phenotype: A second level of stratification may involve biomarkers reflecting BBB integrity and neurovascular dysfunction. Although no single biomarker currently possesses sufficient sensitivity or specificity for clinical implementation, combining circulating BBB-associated proteins, endothelial EVs, CSF markers and advanced neuroimaging may improve the identification of patients with biologically relevant neurovascular impairment. Importantly, BBB dysfunction is unlikely to represent an all-or-none phenomenon but rather a continuum that may fluctuate with inflammatory activity, metabolic status and disease stage (Nation et al., 2019; Knox et al., 2022; Wakonigg Alonso et al., 2024; Zhang et al., 2025).

Mitochondrial phenotype: The third layer may consist of mitochondrial phenotyping. Measurements such as mtDNA copy number, circulating cell-free mitochondrial DNA, OXPHOS capacity, mitochondrial respiration in PBMCs, mitochondrial membrane potential, ROS generation and metabolomic signatures may help distinguish patients in whom mitochondrial dysfunction represents an active biological process rather than a secondary consequence of chronic illness. Future integration of these variables with inflammatory and vascular biomarkers may provide considerably greater biological resolution than any individual marker alone (Picard and McEwen, 2018; Picard and Shirihai, 2022; Bisle et al., 2025; Ye J. et al., 2025).

Systemic metabolic phenotype: Metabolic health represents another clinically meaningful source of heterogeneity. Obesity, insulin resistance, dyslipidemia, hypertension, type 2 diabetes and chronic sleep disruption promote oxidative stress, endothelial dysfunction, mitochondrial remodeling and persistent low-grade inflammation. Rather than acting as independent comorbidities, these systemic disturbances may amplify mitochondrial–BBB vulnerability and contribute to accelerated disease progression, cognitive impairment and reduced treatment responsiveness (Milaneschi et al., 2019; Colombo et al., 2024; Fabbri, 2025). This layer also emphasizes that psychiatric disorders should increasingly be considered systemic conditions in which peripheral metabolic abnormalities interact bidirectionally with brain function.

Clinical phenotype: The final layer integrates clinically relevant outcomes that may emerge from the interaction among the previous biological domains. Cognitive impairment appears particularly informative because BBB dysfunction can impair nutrient transport, glymphatic clearance and neurovascular coupling, whereas mitochondrial dysfunction compromises ATP availability, calcium buffering and synaptic plasticity. Together these mechanisms may contribute to persistent deficits in executive function, attention, processing speed and memory across multiple psychiatric disorders. Similarly, treatment response may represent an important biological dimension. Persistent inflammation, oxidative stress, metabolic disease and mitochondrial dysfunction may reduce therapeutic efficacy by altering neuroplasticity, neurotransmitter metabolism, cerebral perfusion, endothelial transport systems and possibly drug penetration across the BBB. Accordingly, treatment-resistant illness should not necessarily be interpreted solely as insufficient monoaminergic modulation but may instead reflect underlying immune, vascular and metabolic dysregulation requiring broader biological characterization. Recent transcriptomic, imaging and inflammatory studies increasingly support the existence of biologically distinct forms of treatment-resistant psychiatric disorders, although prospective validation remains necessary before these approaches can be incorporated into routine clinical practice (Bravi et al., 2025; Fabbri, 2025; Sirés et al., 2025).

**FIGURE 5:**
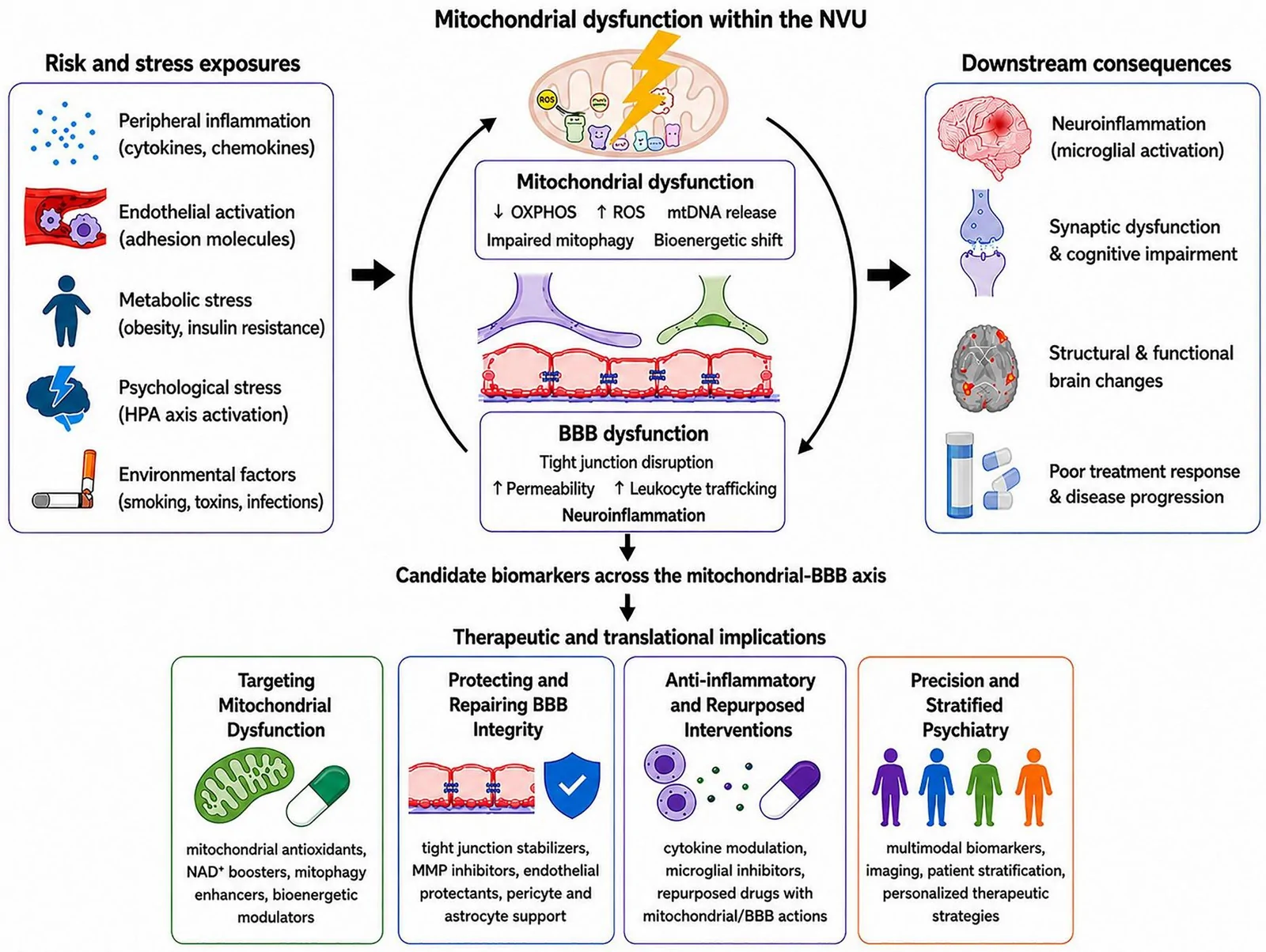
Translational framework for the mitochondrial–blood-brain barrier axis in neuroinflammatory psychiatric disorders. Risk and stress exposures (left, blue outline) are proposed to interact with mitochondrial alterations and blood-brain barrier (BBB) dysfunction (center). Opposing circular arrows indicate a hypothesized bidirectional, self-amplifying relationship rather than proven causality. Black horizontal arrows connect exposures with the central framework and candidate downstream consequences (right, blue outline); the downward arrow leads to candidate biomarkers and therapeutic strategies. Green denotes mitochondrial-targeted strategies, blue BBB-protective or reparative strategies, purple anti-inflammatory or repurposed interventions, and orange precision/stratified psychiatry. HPA, hypothalamic–pituitary–adrenal; MMP, matrix metalloproteinase; mtDNA, mitochondrial DNA; NAD+, oxidized nicotinamide adenine dinucleotide; NVU, neurovascular unit; OXPHOS, oxidative phosphorylation; ROS, reactive oxygen species. ↑ and ↓ indicate relative increases and decreases. No inhibitory bar is used. All links denote testable associations or proposed mechanisms. Left-to-right translational framework. Inflammatory, endothelial, metabolic, psychological, and environmental exposures connect to a central bidirectional cycle of mitochondrial and blood–brain barrier dysfunction. This cycle connects to neuroinflammation, synaptic and structural brain changes, and poor treatment response. A downward branch organizes candidate interventions into mitochondrial targeting, barrier protection, anti-inflammatory or repurposed treatment, and precision-stratified psychiatry.

Integrative perspective: Taken together, these five biological dimensions support a precision psychiatry framework in which inflammation, BBB dysfunction, mitochondrial biology, metabolic health and clinical phenotype are considered complementary rather than competing sources of information. Instead of searching for a single diagnostic biomarker, future studies should aim to integrate these domains into multidimensional biological profiles capable of identifying patients who share common disease mechanisms despite heterogeneous clinical presentations. Such an approach may ultimately facilitate biomarker-guided clinical trials, improve patient stratification, and enable personalized therapeutic strategies targeting specific neuroimmune, metabolic and neurovascular pathways. At present, however, this framework should be considered a research model that requires prospective validation in longitudinal cohorts and multimodal clinical studies before implementation in routine psychiatric practice. The proposed multidomain stratification framework is summarized in Table 3, and its translational context is illustrated in Figure 5.

**TABLE 3:** Translating the mitochondrial–blood-brain barrier axis into precision psychiatry: a proposed multidomain biological framework.

Biological domain	Central biological question	Representative biomarkers	Clinical interpretation	Potential therapeutic opportunities	Current limitations	Future research priorities
Inflammatory domain	Is immune activation a prominent component of the biological profile?	CRP, IL-6, TNF-α, IL-1β, IL-18, MCP-1, kynurenine pathway, TSPO PET	Supports identification of inflammatory endotypes associated with poorer outcomes	Anti-inflammatory and immunometabolic therapies	Limited specificity; inflammatory markers fluctuate over time	Prospective biomarker-guided intervention trials
Neurovascular domain	Is there evidence of BBB or neurovascular vulnerability?	S100B, claudin-5, MMP-9, ICAM-1, VCAM-1, endothelial EVs, QAlb, DCE-MRI	Supports identification of BBB dysfunction or neurovascular vulnerability	BBB stabilization and endothelial-protective therapies	No single validated BBB biomarker	Multimodal BBB assessment combining imaging and fluid biomarkers
Mitochondrial domain	Are mitochondrial abnormalities part of the active biological phenotype?	mtDNAcn, cf-mtDNA, OCR, ΔΨm, mtROS, NAD+/NADH, metabolomics	Supports identification of mitochondrial endotypes	Exercise, NAD+ restoration, mitochondrial antioxidants, mitophagy enhancers	Peripheral markers may not fully reflect brain mitochondria	Validation against CNS and longitudinal outcomes
Systemic metabolic domain	Which systemic factors may amplify mitochondrial–BBB vulnerability?	BMI, HOMA-IR, HbA1c, lipid profile, blood pressure, sleep quality	Defines systemic modifiers of neurovascular vulnerability	Lifestyle and cardiometabolic optimization	Metabolic abnormalities are not disease-specific	Integrate metabolic and neurobiological phenotyping
Clinical domain	How are biological profiles associated with patient outcomes?	Cognition, fatigue, negative symptoms, treatment response	Links biology with clinically meaningful phenotypes	Personalized treatment selection and monitoring	Endotypes remain insufficiently validated	Prospective multimodal precision psychiatry cohorts

BBB, blood-brain barrier; BMI, body mass index; cf-mtDNA, cell-free mitochondrial DNA; CRP, C-reactive protein; DCE-MRI, dynamic contrast-enhanced magnetic resonance imaging; EVs, extracellular vesicles; HbA1c, glycated hemoglobin; HOMA-IR, homeostatic model assessment of insulin resistance; ICAM-1, intercellular adhesion molecule 1; IL, interleukin; MCP-1, monocyte chemoattractant protein 1; MMP-9, matrix metalloproteinase 9; mtDNAcn, mitochondrial DNA copy number; mtROS, mitochondrial reactive oxygen species; NAD+/NADH, oxidized/reduced nicotinamide adenine dinucleotide; OCR, oxygen-consumption rate; QAlb, cerebrospinal fluid/serum albumin quotient; TNF-α, tumor necrosis factor alpha; TSPO, 18-kDa translocator protein; VCAM-1, vascular cell adhesion molecule 1; ΔΨm, mitochondrial membrane potential.

## 8 Future perspectives

The mitochondrial–BBB axis provides a useful framework for connecting systemic metabolic and inflammatory disturbances with neurovascular dysfunction and psychiatric phenotypes. However, future research must move beyond parallel associations to establish temporal relationships, cellular specificity, causality, and treatment responsiveness. Longitudinal and multimodal studies assessing mitochondrial function and BBB integrity in the same individuals are needed to determine whether mitochondrial dysfunction precedes barrier impairment, develops secondary to neurovascular inflammation, or participates in a self-amplifying pathological cycle.

Patient-derived iPSC, multicellular BBB organoids, vascularized brain organoids, and BBB-on-chip platforms may help reproduce the cellular complexity of the NVU while accounting for psychiatric heterogeneity. These models could integrate genetic perturbations, mitochondrial haplotypes and heteroplasmy, and exposure to patient-derived serum or immune cells. Mitochondrial characterization should include respiration, membrane potential, redox balance, calcium handling, dynamics, mitophagic flux, and mtDNA release rather than relying exclusively on oxidative-stress markers.

Human studies should combine mitochondrial biomarkers—including circulating mtDNA, NAD+/NADH balance, acylcarnitines and PBMC bioenergetics—with inflammatory, endothelial, and BBB-related measures. Integration with DCE-MRI, ASL, cerebrovascular-reactivity testing, MRS, or the QAlb could provide stronger evidence of neurovascular involvement. Repeated assessments across acute episodes, remission, relapse, and treatment would help distinguish vulnerability traits from state-related, medication-induced, or metabolically driven changes.

The mitochondrial–BBB axis should also be investigated across disorders and transdiagnostic dimensions. In MDD, inflammatory and metabolically impaired subgroups may show the strongest associations with fatigue, anhedonia, and cognitive dysfunction. In PTSD, prospective studies following trauma could clarify how stress hormones, sympathetic activation, sleep disturbance, and inflammation affect mitochondrial and vascular function. Developmental and genetic-risk models may be particularly informative in SZ-spectrum disorders, whereas illness stage, circadian disruption, and metabolic comorbidity should be considered in BD and ASD.

Therapeutic research should use staged, mechanism-informed trials. Interventions such as MitoQ, elamipretide, CoQ10, NAD+ enhancers, melatonin, N-acetylcysteine, mitophagy activators, and PGC-1α-directed strategies should first demonstrate mitochondrial target engagement and then determine whether this produces measurable improvements in BBB/NVU function and clinical outcomes. Biomarker-enriched or adaptive trials could match therapies to inflammatory, redox, bioenergetic, mitophagic, metabolic, circadian, or vascular profiles rather than applying them to unselected diagnostic groups.

Finally, standardized preanalytical procedures and convergent biomarker approaches will be essential because peripheral mitochondrial and BBB-related measures are indirect and sensitive to medication, metabolic disease, exercise, infection, and sample handling. Future studies should also distinguish adaptive mitohormesis from chronic maladaptive mitochondrial stress. Defining this context-dependent threshold may explain the inconsistent effects of broad antioxidant interventions and identify the therapeutic window in which mitochondrial modulation restores BBB and neurovascular resilience.

## 9 Conclusion

Mitochondrial dysfunction and BBB dysregulation may form a convergent interface through which inflammation, metabolic stress, and neurovascular vulnerability relate to psychiatric phenotypes. This is neither a replacement for diagnosis nor a universal causal mechanism. Instead, it may define subgroups characterized by inflammatory activation, mitochondrial stress, BBB vulnerability, cognitive or motivational symptoms, and poor treatment response. Multimodal longitudinal studies should test whether mitochondrial, anti-inflammatory, metabolic, or BBB-protective interventions benefit specific biological subgroups.
