# Supplementary material for: Mitochondrial control of blood-brain barrier homeostasis in neuroinflammatory psychiatric disorders
Source: Front Mol Neurosci. 2026 Aug 24;19:1924581. doi: 10.3389/fnmol.2026.1924581 (PMC13548091; doi:10.3389/fnmol.2026.1924581)
Supplement: Supplementary file 1 [file Data_Sheet_1.docx]

**Supplementary table 1. Evidence for mitochondrial and BBB alterations across neuroinflammatory psychiatric disorders.**

| **Disorder/ Mechanism** | **Models / cohorts supporting** | **Mitochondrial alterations** | **BBB / neurovascular** | **Inflammatory alterations** | **Integrated interpretation** | **Key References** |
| --- | --- | --- | --- | --- | --- | --- |
| **SZ** | Postmortem brain, CSF studies, DCE-MRI, iPSC-derived BBB models, vascular organoids | Impaired mitochondrial bioenergetics, endothelial metabolic vulnerability, altered mitochondrial redox regulation | ↓ CLDN5, altered CLDN12 and ZO-1, ↑ CSF/serum albumin ratio, ↑ BBB permeability, abnormal angiogenesis | ↑ IL-6, ↑ IL-8, endothelial activation, peripheral cytokine dysregulation | Endothelial mitochondrial alterations are associated with BBB instability and chronic neuroimmune activation; causality has not been established in patients. | Greene et al., 2020; Stankovic et al., 2024; Moussiopoulou et al., 2025 |
| **MDD** | Clinical cohorts, PBMC studies, postmortem tissue, chronic social defeat stress models | ↑ cf-mtDNA, altered mitochondrial respiration, oxidative stress, impaired bioenergetic adaptation to stress | ↓ Claudin-5, BBB leakage in nucleus accumbens and hippocampus, altered VCAM-1 signaling, endothelial dysfunction | ↑ IL-6, ↑ TNF-α, ↑ IL-1β, ↑ CRP, ↑ CCL2/MCP-1; stress-induced immune activation | Human findings support associations among chronic stress, mitochondrial alterations and BBB-related abnormalities; preclinical models suggest mechanisms by which inflammatory mediators may gain brain access. | Lindqvist et al., 2018; Menard et al., 2017; Dudek et al., 2020; Dion-Albert et al., 2022; Köhler-Forsberg et al., 2017 |
| **BD** | CSF studies, neuroimaging, spectroscopy, biomarker studies | ↑ CSF lactate, ETC/complex I dysfunction, altered oxidative metabolism, redox imbalance | Altered neurovascular coupling, increased S100B, albumin ratio, cell-adhesion molecules, MMPs, and claudin-5 alterations | Episodic increases in TNF-α, IL-1β, IL-18, NF-κB and NLRP3-related signaling | Mitochondrial dysfunction and inflammatory signaling may contribute to BBB vulnerability, particularly during acute mood episodes. | Regenold et al., 2008; Futtrup et al., 2020; Jones et al., 2021 |
| **ASD** | Postmortem brain, intestinal biopsies, fibroblasts, lymphoblastoid cells, developmental cohorts | Altered mitochondrial morphology, ETC dysfunction, respiratory-chain uncoupling, altered lactate, pyruvate and acylcarnitine metabolism | Altered CLDN3, CLDN5, CLDN12, tricellulin and MMP-9 expression; intestinal barrier disruption with loss of tight-junction proteins | Neuroinflammation, oxidative stress susceptibility, immune-metabolic dysregulation | ASD appears to include a mitochondrial-neuroimmune subtype characterized by abnormal barrier development and increased susceptibility to inflammatory signaling. | Fiorentino et al., 2016; Rossignol & Frye, 2011; Rose et al., 2014; Frye et al., 2021; Frye et al., 2024 |
| **PTSD / trauma-related disorders** | Combat veterans, plasma biomarker studies, chronic stress models | ↓ mtDNA copy number, ↑ circulating cell-free mtDNA, altered mitochondrial stress responses | Direct BBB evidence is limited, but stress models show endothelial dysfunction and BBB vulnerability | HPA-axis dysregulation, sterile inflammation, stress-associated cytokine activation | Severe stress is associated with mitochondrial alterations and circulating mtDNA; experimental evidence suggests that mtDNA danger signaling may amplify inflammatory responses. | Bersani et al., 2016; Lindqvist et al., 2016; Blalock et al., 2024 |
| **Transdiagnostic neurovascular mechanism** | Endothelial cultures, BBB models, hypoxia models, inflammatory models, stroke/sepsis models | ↓ OXPHOS, mitochondrial fragmentation (DRP1/Fis1), mtDNA release, defective mitophagy (PINK1/Parkin), ↑ mitochondrial ROS | Loss of ZO-1, occludin and claudin-5 integrity; ↑ permeability; altered GLUT1, LAT1 and P-gp transporters; endothelial activation | ↑ NF-κB, ↑ NLRP3, ↑ ICAM-1, ↑ VCAM-1, ↑ IFN-I signaling, leukocyte adhesion | In experimental systems, mitochondrial dysfunction can induce BBB disruption through oxidative stress, inflammasome activation and endothelial inflammatory reprogramming; direct clinical causality remains unproven. | Doll et al., 2015; Haileselassie et al., 2020; Nakahira et al., 2011; Shimada et al., 2012; Guo et al., 2020; Huang et al., 2020 |
| **NVU cell-specific mechanisms** | Astrocyte-, microglia- and pericyte-based models | Astrocytic mitochondrial support, pericyte mitophagy defects, microglial metabolic reprogramming and mitochondrial ROS production | Pericyte loss, astrocytic endfoot disruption, microglial-mediated BBB remodeling, impaired capillary stability and cerebral blood flow regulation | NLRP3 activation, SPP1 signaling, IL-1β release, phagocytic microglial states | Cell-specific models suggest that mitochondrial dysfunction may impair NVU communication and promote BBB destabilization and neuroinflammation. | Daneman et al., 2010; Haruwaka et al., 2019; De Schepper et al., 2023; Li et al., 2022; Baik et al., 2019 |

**Abbreviations:** ASD, autism spectrum disorder; BBB, blood–brain barrier; BD, bipolar disorder; CCL2, C-C motif chemokine ligand 2; cf-mtDNA, cell-free mitochondrial DNA; CLDN3/5/12, claudin-3/5/12; CRP, C-reactive protein; CSF, cerebrospinal fluid; DAMP, damage-associated molecular pattern; DCE-MRI, dynamic contrast-enhanced magnetic resonance imaging; DRP1, dynamin-related protein 1; ETC, electron transport chain; Fis1, mitochondrial fission 1 protein; GLUT1, glucose transporter 1; HPA, hypothalamic–pituitary–adrenal; ICAM-1, intercellular adhesion molecule 1; IFN-I, type I interferon; IL, interleukin; iPSC, induced pluripotent stem cell; LAT1, L-type amino acid transporter 1; MCP-1, monocyte chemoattractant protein 1; MDD, major depressive disorder; MMP, matrix metalloproteinase; mtDNA, mitochondrial DNA; NF-κB, nuclear factor kappa B; NLRP3, NOD-, LRR- and pyrin domain-containing protein 3; NVU, neurovascular unit; OXPHOS, oxidative phosphorylation; PBMC, peripheral blood mononuclear cell; P-gp, P-glycoprotein; PINK1, PTEN-induced kinase 1; PTSD, post-traumatic stress disorder; ROS, reactive oxygen species; SPP1, secreted phosphoprotein 1; SZ, schizophrenia; TNF-α, tumor necrosis factor alpha; VCAM-1, vascular cell adhesion molecule 1; ZO-1, zonula occludens 1.

**Supplementary table 2. Candidate biomarkers for the mitochondrial–BBB axis**

| **Domain** | **Biomarker** | **Sample** | **Interpretation** | **Caveat** | **Key references** |
| --- | --- | --- | --- | --- | --- |
| **Peripheral inflammatory activation** | CRP + IL-6 + TNF-α + IL-1β | Serum/plasma | Reflects systemic inflammatory burden associated with endothelial activation, BBB vulnerability, cognitive impairment and treatment resistance. Useful for identifying inflammatory psychiatric subgroups. | Highly influenced by obesity, smoking, infection, metabolic syndrome and medication exposure; low CNS specificity. | Raison et al., 2013; Boozalis et al., 2018; Sæther et al., 2022; Chumakov et al., 2022; Poletti et al., 2024 |
| **Endothelial immune activation** | CCL2/MCP-1 + sICAM-1 + sVCAM-1 | Serum/plasma; CSF | Indicates leukocyte recruitment, endothelial activation and early neurovascular dysfunction. Preclinical evidence links VCAM-1 signaling to stress-associated BBB disruption and depressive-like behavior. | Can originate from peripheral vasculature; should be interpreted alongside BBB-specific markers. | Dion-Albert et al., 2022; Meixensberger et al., 2021; Sheikh et al., 2023 |
| **BBB permeability** | CSF/serum albumin quotient | Paired CSF + serum | Current clinical gold standard for assessing barrier permeability; elevated values indicate passive influx of circulating proteins into CNS compartments. | Invasive procedure; reflects BBB and blood–CSF barrier integrity simultaneously. | Meixensberger et al., 2020; Konstantinou & Warsh, 2026 |
| **Astroglial–vascular injury** | S100B ± Neurofilament light chain (NfL) | Serum/plasma; CSF | Reflects astrocytic stress, neurovascular injury and possible BBB dysfunction. Combined S100B/NfL panels may improve prediction of neuroprogression. | S100B is not BBB-specific and may originate from peripheral tissues. | Futtrup et al., 2020; Kozlowski et al., 2023; Comai, 2025 |
| **Tight-junction integrity** | Claudin-5 + Occludin + ZO-1 | Serum/plasma; tissue; BBB models | Reflects structural BBB integrity. Reduced tight-junction proteins are among the strongest indicators of physical barrier disruption. | Peripheral quantification remains insufficiently standardized. | Jiao et al., 2011; Greene et al., 2020; Stankovic et al., 2024; Sun et al., 2025 |
| **Extracellular matrix remodeling** | MMP-9 + MMP-2 | Serum/plasma; CSF | Indicates inflammatory proteolysis, extracellular-matrix remodeling and tight-junction cleavage associated with BBB opening. | Better interpreted as remodeling markers than direct measures of permeability. | Feng et al., 2011; Futtrup et al., 2020; Greene et al., 2020; Sun et al., 2025 |
| **Cell-specific neurovascular liquid biopsy** | Endothelial- and astrocyte-derived EVs | Plasma/serum | Provide cell-specific cargo including proteins, miRNAs, inflammasome mediators, complement components and mitochondrial molecules, offering a minimally invasive window into neurovascular pathology. | Isolation and normalization methods remain heterogeneous. | Comai, 2025; Ye et al., 2025 |
| **Mitochondrial DNA dynamics** | cf-mtDNA + mtDNA copy number | Plasma, serum, CSF, whole blood, PBMCs | Reflect mitochondrial injury, DAMP signaling, mitochondrial biogenesis and chronic bioenergetic adaptation. cf-mtDNA may directly activate cGAS–STING, TLR9 and inflammasome pathways. | Strongly influenced by sample processing, platelet contamination, cellular composition and disease stage. | Lindqvist et al., 2016; Lindqvist et al., 2018; Kumar et al., 2018; Guo et al., 2020; Huang et al., 2020; Ouyang et al., 2021; Blalock et al., 2024 |
| **Bioenergetic metabolism** | Lactate, pyruvate and lactate/pyruvate ratio | Plasma; CSF; MRS | Reflects impaired oxidative phosphorylation and compensatory glycolytic metabolism. Elevated ratios suggest mitochondrial inefficiency and altered cerebral energy metabolism. | Influenced by exercise, hypoxia and systemic metabolic conditions. | Regenold et al., 2008; Dwir et al., 2019; Giménez-Palomo et al., 2024 |
| **Mitochondrial functional profile** | OCR/OXPHOS capacity + ΔΨm + mitochondrial ROS | PBMCs; endothelial cells; BBB models | Direct assessment of mitochondrial respiration, membrane polarization and oxidative stress burden. Integrates the principal functional dimensions of mitochondrial health relevant to BBB maintenance. | Requires viable cells, specialized platforms and methodological standardization. | Schreibelt et al., 2007; Doll et al., 2015; Hu et al., 2019; Haileselassie et al., 2020; Kim et al., 2022; Weber et al., 2022; Wang et al., 2023 |
| **Mitochondrial quality control** | PINK1, Parkin, LC3-II/p62 ratio, mitophagy-related markers | PBMCs; EVs; experimental models | Reflects mitophagy efficiency and mitochondrial quality-control mechanisms. Experimental evidence associates defective mitophagy with ROS accumulation, inflammasome activation, endothelial dysfunction and BBB vulnerability. | Human psychiatric evidence remains limited and assay standardization is lacking. | Nakahira et al., 2011; Wu et al., 2015 |
| **Immunometabolic dysfunction** | NAD⁺/NADH metabolites + kynurenine pathway metabolites | Plasma; PBMCs; metabolomics | Reflects coupling between inflammation, mitochondrial dysfunction, redox imbalance and neurovascular vulnerability. May identify biologically distinct patient subgroups. | Peripheral measurements may not accurately represent CNS target engagement. | Dwir et al., 2019; Cuklanz et al., 2026 |
| **In vivo neurovascular phenotype** | DCE-MRI BBB leakage | Brain MRI | Quantitative measure of regional BBB permeability and neurovascular dysfunction in living patients. Represents the most direct non-invasive assessment of BBB integrity. | Expensive, technically demanding and not suitable for routine screening. | Moussiopoulou et al., 2025 |

**Abbreviations:** BBB, blood–brain barrier; CCL2, C-C motif chemokine ligand 2; cf-mtDNA, cell-free mitochondrial DNA; cGAS–STING, cyclic GMP–AMP synthase–stimulator of interferon genes; CNS, central nervous system; CRP, C-reactive protein; CSF, cerebrospinal fluid; DAMP, damage-associated molecular pattern; DCE-MRI, dynamic contrast-enhanced magnetic resonance imaging; EVs, extracellular vesicles; ICAM-1, intercellular adhesion molecule 1; IL, interleukin; LC3-II, microtubule-associated protein 1A/1B-light chain 3-II; MCP-1, monocyte chemoattractant protein 1; miRNAs, microRNAs; MMP, matrix metalloproteinase; MRS, magnetic resonance spectroscopy; mtDNA, mitochondrial DNA; NAD+/NADH, oxidized/reduced nicotinamide adenine dinucleotide; NfL, neurofilament light chain; OCR, oxygen consumption rate; OXPHOS, oxidative phosphorylation; PBMCs, peripheral blood mononuclear cells; PINK1, PTEN-induced kinase 1; ROS, reactive oxygen species; S100B, S100 calcium-binding protein B; sICAM-1, soluble intercellular adhesion molecule 1; sVCAM-1, soluble vascular cell adhesion molecule 1; TLR9, Toll-like receptor 9; TNF-α, tumor necrosis factor alpha; VCAM-1, vascular cell adhesion molecule 1; ZO-1, zonula occludens 1; ΔΨm, mitochondrial membrane potential.

# Supplementary search strategies

Database: PubMed. Last search: 25 July 2026. Language: English. The searches were performed independently and their results were screened together. Because this was a narrative review, the searches were designed for sensitivity and conceptual coverage rather than as a systematic-review protocol.

**Strategy 1. Core mitochondrial–blood–brain barrier/neurovascular-unit axis**

(("Mitochondria"[Mesh] OR mitochondri*[Title/Abstract] OR "mitochondrial dysfunction"[Title/Abstract] OR mitophagy[Title/Abstract] OR "oxidative phosphorylation"[Title/Abstract] OR OXPHOS[Title/Abstract] OR "mitochondrial reactive oxygen species"[Title/Abstract] OR mtROS[Title/Abstract] OR "mitochondrial DNA"[Title/Abstract] OR mtDNA[Title/Abstract]) AND ("Blood-Brain Barrier"[Mesh] OR "blood-brain barrier"[Title/Abstract] OR BBB[Title/Abstract] OR "neurovascular unit"[Title/Abstract] OR "brain endothelial"[Title/Abstract] OR "cerebral endothelial"[Title/Abstract]) AND (dysfunction*[Title/Abstract] OR disrupt*[Title/Abstract] OR permeab*[Title/Abstract] OR integrity[Title/Abstract] OR inflammation[Title/Abstract] OR oxidative[Title/Abstract])) AND (english[Language]) AND ("1900/01/01"[Date - Publication] : "2026/07/25"[Date - Publication])

**Strategy 2. Psychiatric-disorder evidence**

(("Mitochondria"[Mesh] OR mitochondri*[Title/Abstract] OR mitophagy[Title/Abstract] OR mtDNA[Title/Abstract] OR bioenergetic*[Title/Abstract]) AND ("Blood-Brain Barrier"[Mesh] OR "blood-brain barrier"[Title/Abstract] OR BBB[Title/Abstract] OR "neurovascular unit"[Title/Abstract] OR neurovascular[Title/Abstract] OR endothelial[Title/Abstract]) AND ("Mental Disorders"[Mesh] OR psychiatr*[Title/Abstract] OR schizophrenia[Title/Abstract] OR psychosis[Title/Abstract] OR "major depressive disorder"[Title/Abstract] OR depression[Title/Abstract] OR "bipolar disorder"[Title/Abstract] OR "post-traumatic stress disorder"[Title/Abstract] OR PTSD[Title/Abstract] OR "autism spectrum disorder"[Title/Abstract] OR autism[Title/Abstract])) AND (english[Language]) AND ("1900/01/01"[Date - Publication] : "2026/07/25"[Date - Publication])

**Strategy 3. Mitochondria-targeted and related therapeutic approaches**

(("Blood-Brain Barrier"[Mesh] OR "blood-brain barrier"[Title/Abstract] OR BBB[Title/Abstract] OR "neurovascular unit"[Title/Abstract] OR "brain endothelial"[Title/Abstract]) AND ("Mitochondria"[Mesh] OR mitochondri*[Title/Abstract] OR mitophagy[Title/Abstract] OR NAD+[Title/Abstract] OR "mitochondrial biogenesis"[Title/Abstract]) AND (MitoQ[Title/Abstract] OR elamipretide[Title/Abstract] OR SS-31[Title/Abstract] OR "coenzyme Q10"[Title/Abstract] OR ubiquinone[Title/Abstract] OR "nicotinamide riboside"[Title/Abstract] OR "nicotinamide mononucleotide"[Title/Abstract] OR melatonin[Title/Abstract] OR "N-acetylcysteine"[Title/Abstract] OR NAC[Title/Abstract] OR "mitophagy activator"[Title/Abstract] OR "mitophagy activation"[Title/Abstract] OR "PGC-1alpha"[Title/Abstract] OR "PGC-1α"[Title/Abstract] OR "NAD+ booster"[Title/Abstract])) AND (english[Language]) AND ("1900/01/01"[Date - Publication] : "2026/07/25"[Date - Publication])
